# Multiplexed In Vivo Imaging with Fluorescence Lifetime‐Modulating Tags

**DOI:** 10.1002/advs.202404354

**Published:** 2024-06-20

**Authors:** Lina El Hajji, France Lam, Maria Avtodeeva, Hela Benaissa, Christine Rampon, Michel Volovitch, Sophie Vriz, Arnaud Gautier

**Affiliations:** ^1^ Sorbonne Université École Normale Supérieure Université PSL CNRS Laboratoire des Biomolécules LBM Paris 75005 France; ^2^ Institut de Biologie Paris Seine plateforme imagerie photonique I2PS (FR3631) Sorbonne Université CNRS Paris 75005 France; ^3^ Université Paris Cité Paris 75006 France; ^4^ Institut Universitaire de France Paris 75006 France

**Keywords:** bioimaging, fluorescence lifetime imaging, fluorescent probes, multiplexed imaging, protein engineering

## Abstract

Fluorescence lifetime imaging microscopy (FLIM) opens new dimensions for highly multiplexed imaging in live cells and organisms using differences in fluorescence lifetime to distinguish spectrally identical fluorescent probes. Here, a set of fluorescence‐activating and absorption‐shifting tags (FASTs) capable of modulating the fluorescence lifetime of embedded fluorogenic 4‐hydroxybenzylidene rhodanine (HBR) derivatives is described. It is shown that changes in the FAST protein sequence can vary the local environment of the chromophore and lead to significant changes in fluorescence lifetime. These fluorescence lifetime‐modulating tags enable multiplexed imaging of up to three targets in one spectral channel using a single HBR derivative in live cells and live zebrafish larvae. The combination of fluorescence lifetime multiplexing with spectral multiplexing allows to successfully image six targets in live cells, opening great prospects for multicolor fluorescence lifetime multiplexing.

## Graphical abstract

Fluorescence lifetime‐modulating tags enable the imaging of several cellular targets in a single spectral channel, opening great prospects for highly multiplexed imaging in live cells and organisms.

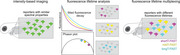

## 1 Introduction

Fluorescence lifetime imaging microscopy (FLIM) has recently gathered broad attention in biological imaging thanks to the development of easy‐to‐use acquisition setups and approaches for data analysis.^[^ ^1^ ^]^ FLIM leverages the fluorescence lifetime, i.e., the average duration of the excited state, of a fluorescent species for probing a given biological sample. Characterization of the lifetime properties of commonly used fluorescent reporters allowed the use of FLIM in a variety of biological contexts. Combined with Förster Resonance Energy Transfer (FRET), FLIM enables detection of protein‐protein interactions through changes in the donor fluorescence lifetime.^[^ ^2^ ^]^ Engineering of fluorescent biosensors with FLIM responsiveness allowed for quantitative imaging of biomolecules of interest through measurements of fluorescence lifetime instead of signal intensity.^[^ ^3^ ^]^

One interesting use of FLIM is the possibility to simultaneously visualize several cellular targets relying on their fluorescence lifetime. Multiplexed observation is usually achieved by multicolor fluorescence microscopy using fluorescent tags displaying different spectral properties (different “colors”). The wide excitation and emission spectra of most fluorophores limit, however, the number of species that can be imaged simultaneously in fluorescence microscopy to four or five. Recently, FLIM based on time‐correlated single photon counting (TCSPC) has gathered attention as it opens additional discrimination dimension to distinguish fluorophores with similar colors.^[^ ^4^ ^]^ Visualization of up to nine fluorescent proteins has been recently achieved using multicolor FLIM,^[^ ^5^ ^]^ demonstrating the potential of FLIM for highly multiplexed imaging using readily available fluorescent reporters. To expand the possibilities offered by fluorescence lifetime multiplexing, dedicated engineering strategies have been developed using lifetime as a criterium to identify fluorescent proteins^[^ ^3^, ^6^ ^]^ and HaloTag‐based chemogenetic fluorescent reporters^[^ ^7^ ^]^ that display a wider range of fluorescence lifetime differences.

Here, we describe the identification of variants of the fluorescence‐activating and absorption‐shifting tag (FAST) that are able to modulate the fluorescence lifetime of embedded 4‐hydroxybenzylidene rhodanine (HBR) derivatives for fluorescence lifetime multiplexing in live cells and organisms. Small proteins of only 14 kDa, FAST and its derivatives are attractive alternatives to fluorescent proteins and self‐labeling tags for monitoring gene expression and protein localization with minimal perturbation.^[^ ^8^, ^9^ ^]^ HBR derivatives only fluoresce when embedded within FAST, enabling wash‐free imaging with very high contrast in cells and in multicellular organisms. FASTs have been shown to excel for imaging proteins in anaerobic organisms.^[^ ^10^ ^]^ and to allow imaging of highly dynamic processes thanks to the extremely rapid formation of the fluorescent assembly.^[^ ^11^ ^]^ Here, we show that screening of a collection of FAST variants allowed us to identify three variants that can modulate the local chromophore environment, leading to significant change in the fluorescence lifetime of the embedded chromophore. These fluorescence lifetime‐modulating tags allow the imaging of three targets in one spectral channel using a single fluorogenic chromophore in live mammalian cells and in zebrafish larvae, opening great prospects for highly multiplexed imaging.

## 2 Results

### 2.1 Identification and Characterization of FAST Variants for FLIM Multiplexing

4‐Hydroxybenzylidene rhodanine (HBR) derivatives are push‐pull chromophores with fluorescence properties highly dependent on the environment. When free, they are almost non‐fluorescent, as they dissipate light energy through ultrafast non‐radiative de‐excitation pathways. Rotations around the methylene bridge bonds have been proposed to promote internal conversion by conical intersection. When embedded within FAST (fluorescence‐activating and absorption‐shifting tag), their anionic state is stabilized and locked in a planar conformation, leading to fluorescence. Different FAST variants adapted to imaging in different biological contexts have been developed over the last few years, mainly through rational design,^[^ ^12^ ^]^ and directed evolution.^[^ ^9^, ^13^ ^]^ Recently, the use of proteins with homology relationship enabled the engineering of new variants, with sequence similarity between 70% to 78% relative to prototypical FAST, all binding prototypical fluorogens with good affinity and yielding fluorescent assemblies with good brightness in vitro and in cells.^[^ ^14^ ^]^ Altogether, these different engineering efforts provide a collection of proteins with a wide diversity of sequences, leading to different photophysical behaviors and thus hypothetically to different fluorescence lifetimes.

**Figure 1:**
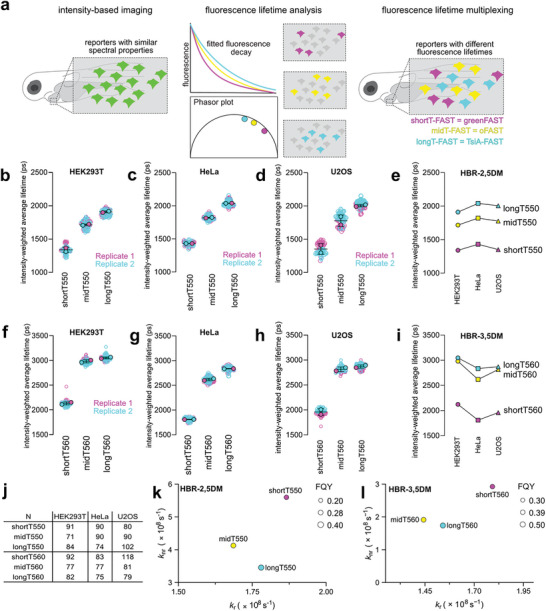
Fluorescence lifetime‐modulating tags. a) Principle of fluorescence lifetime multiplexing of FAST variants. FAST:fluorogen assemblies with similar spectral properties but different lifetime signatures can be distinguished by analyzing their lifetimes. b–e) Intensity‐weighted average lifetime distributions of shortT550, midT550 and longT550 in HEK293T cells b), HeLa cells c) and U2OS cells d). N cells from two biological replicates were analyzed (the number N of analyzed cells is given in j)). Each cell is color‐coded according to the biological replicate it came from. The solid circles correspond to the mean of each biological replicate. The black line represents the mean ± SD of the two biological replicates. e) Means of intensity‐weighted average lifetimes of shortT550, midT550, and longT550 in the three cell lines. f–i) Intensity‐weighted average lifetime distributions of shortT560, midT560, and longT560 in HEK293T cells f), HeLa cells g), and U2OS cells h). N cells from two biological replicates were analyzed (the number N of analyzed cells is given in j)). Each cell is color‐coded according to the biological replicate it came from. The solid circles correspond to the mean of each biological replicate. The black line represents the mean ± SD of the two biological replicates. i) Means of intensity‐weighted average lifetimes of shortT560, midT560, and longT560 in the three cell lines. j) Number of cells for each set of experiments. k,l) Photophysical properties of FAST variants with HBR‐2,5DM k) and HBR‐3,5DM l). Each dot corresponds to one FAST:fluorogen assembly according to their radiative and non‐radiative decay constant values and is scaled to the FQY of the assembly.

To identify FAST:fluorogen assemblies suitable for fluorescence lifetime multiplexing (**Figure** **1**a), we screened fluorogens able to form tight and bright fluorescent assemblies with the largest possible number of variants. We tested the fluorogens HMBR, HBR‐3,5DM and HBR‐2,5DM, which form tight (*K*_D_ 10–900 nM) and bright (fluorescence quantum yield (FQY) between 20%–50%) green‐yellow fluorescent assemblies with the original FAST^[^ ^8^ ^]^ (engineered from the photoactive yellow protein (PYP) from *Halorhodospira halophila*), its variants, iFAST,^[^ ^12^ ^]^ pFAST,^[^ ^9^ ^]^ oFAST,^[^ ^9^ ^]^ tFAST,^[^ ^9^ ^]^ and greenFAST,^[^ ^13^ ^]^ as well as the six recently‐described FAST systems, HboL‐FAST, HspG‐FAST, RspA‐FAST, Ilo‐FAST, TsiA‐FAST, and Rsa‐FAST,^[^ ^14^ ^]^ engineered from six homologs of PYP from *Halomonas boliviensis* LC1 (HboL), *Halomonas* sp. GFAJ‐1 (HspG), *Rheinheimera* sp. A13L (RspA), *Idiomarina loihiensis* (Ilo), *Thiorhodospira sibirica* ATCC 700588 (TsiA) and *Rhodothalassium salexigens* (Rsa) (Text S1 and Figure S1, Supporting Information). We imaged each variant:fluorogen assembly in cultured mammalian cells by TCSPC‐FLIM (Figure S2, Supporting Information). For the screening, we fitted the fluorescence decays of each assembly with a biexponential model and computed the intensity‐weighted average fluorescence lifetime (T_i_) (Figure S2a–c, Supporting Information). The use of T_i_ enables to compare systems regardless of their mono or biexponential decay behavior. In agreement with their relative fluorescence quantum yields, for a given variant, the T_i_ of the fluorescent assembly gradually increased going from HMBR to HBR‐2,5DM and HBR‐3,5DM. Interestingly, with the same fluorogen, although displaying very similar fluorescence quantum yields, some FAST variants yielded fluorescent assemblies with clearly distinguishable lifetime signatures. This behavior was already reported for greenFAST and iFAST, which form assemblies with HMBR having similar fluorescence quantum yields (23% and 22% respectively), but distinguishable T_i_ values (respectively T_i_ = 1.25 ± 0.01 and 1.73 ± 0.01 ns).^[^ ^13^ ^]^ Noteworthily, regardless of the fluorogen used, greenFAST was always the variant yielding the lowest T_i_, while TsiA‐FAST always gave the highest T_i_ (Figure S2d, Supporting Information). Computing of pairwise lifetime differences enabled the identification of variants suitable for fluorescence lifetime multiplexing. Regardless of the fluorogen, greenFAST displays lifetime difference large enough with most FAST variants for multiplexing by FLIM (ΔT_i_ > 0.4 ns), the best discrimination being observed with TsiA‐FAST. greenFAST and TsiA‐FAST displayed a ΔT_i_ of 0.78 ns with HMBR, 0.89 ns with HBR‐2,5DM, and 1.17 ns with HBR‐3,5DM. We identified a third variant, oFAST, that forms fluorescent assemblies with fluorescence lifetimes in between those obtained with greenFAST and TsiA‐FAST (Figure S2, Supporting Information), opening exciting prospects for fluorescence lifetime multiplexing of three FAST variants in the same spectral channel. In this screening, HBR‐2,5DM appeared as the optimal fluorogen for lifetime multiplexing of the three FAST variants, while HBR‐3,5DM offered the best lifetime differences for the separation of two FAST variants. Hereafter, greenFAST, oFAST, and TsiA‐FAST are renamed shortT‐FAST, midT‐FAST, and longT‐FAST, respectively, in which the prefix indicates whether they give short, intermediate, or long fluorescence lifetimes. Moreover, when designating their assemblies with HBR‐2,5DM (resp. HBR‐3,5DM), which emits at about 550 nm (resp. 560 nm) within FAST variants, we will use the names shortT550, midT550, and longT550 (resp. shortT560, midT560 and longT560).

We next performed an extensive characterization of shortT‐FAST, midT‐FAST, and longT‐FAST with HBR‐2,5DM and HBR‐3,5DM in three different mammalian cell lines, HEK293T, HeLa, and U2OS cells (Figure 1b–l; Figures S3‐S7, Supporting Information). The three variants were expressed as fusions to the histone H2B. We fitted the fluorescence decays with either a monoexponential or a biexponential model and determined the best fit model using a reduced χ^2^ goodness‐of‐fit test (Figures S6 and S7, Supporting Information) The variants shortT550 (a.k.a shortT‐FAST:HBR‐2,5DM) and shortT560 (a.k.a. short‐FAST:HBR‐3,5DM) clearly displayed biexponential fluorescence decay. The variants midT550 (a.k.a. midT‐FAST:HBR‐2,5DM) and longT550 (a.k.a. longT‐FAST:HBR‐2,5DM) displayed rather biexponential decay. For midT560 (a.k.a. midT‐FAST:HBR‐3,5DM) and longT560 (a.k.a. longT‐FAST:HBR‐3,5DM), the goodness‐of‐fit with a monoexponential fit model and a biexponential fit model were very similar. To analyze all the systems the same way and facilitate comparison, we use hereafter T_i_ computed from the results of the biexponential fit. For a given variant, comparable T_i_ values were obtained in the three tested cell lines (Figure 1b–d, f–h, j,f), suggesting that this parameter is robust in various cellular contexts. This study confirmed that, for a given fluorogen, the assemblies with shortT‐FAST, midT‐FAST, and longT‐FAST display distinguishable fluorescence lifetimes. With HBR‐2,5DM, the three variants show clearly different lifetime signatures in the three cell lines. With HBR‐3,5DM, the lifetime difference of the pairs shortT‐FAST/midT‐FAST and shortT‐FAST/longT‐FAST was larger than with HBR‐2,5DM in all cell lines. However, with HBR‐3,5DM, the pair midT‐FAST/longT‐FAST displays a lifetime difference smaller than with HBR‐2,5DM in HEK293T and U2OS cells, making their discrimination in these cell lines more challenging. In HeLa cells, shortT‐FAST and midT‐FAST display a difference of lifetime ΔT_i_ = 0.39 ns with HBR‐2,5DM and ΔT_i_ = 0.80 ns with HBR‐3,5DM. midT‐FAST and longT‐FAST display a difference of lifetime ΔT_i_ = 0.22 ns with HBR‐2,5DM and ΔT_i_ = 0.22 ns with HBR‐3,5DM. Finally, shortT‐FAST and longT‐FAST display a difference of lifetime ΔT_i_ = 0.61 ns with HBR‐2,5DM and ΔT_i_ = 1.0 ns with HBR‐3,5DM. For each variant, the T_i_ values measured in the three different cell lines were close (Figure 1e,i), demonstrating the possibility to distinguish them regardless of the cell type used. We assume that the ability of the three variants to modulate differently the fluorescence lifetime of the embedded fluorogen is related to their different amino acid sequences that modulate the local environment of the chromophore, affecting thus the fate of the excited state. shortT‐FAST and midT‐FAST share 94% sequence identity, shortT‐FAST and longT‐FAST 71%, and midT‐FAST and longT‐FAST 74%. Estimation of the kinetic constants associated with radiative and non‐radiative de‐excitation suggested that the differences in fluorescence lifetime we observed are mainly due to differences in the non‐radiative kinetic constants (Figure 1k,l). While longT‐FAST and midT‐FAST display close radiative and non‐radiative constants regardless of the fluorogen, shortT‐FAST is characterized by a higher non‐radiative kinetic constant compared to the two other reporters.

### 2.2 Fluorescence Lifetime Multiplexing in Mammalian Cells

**Figure 2:**
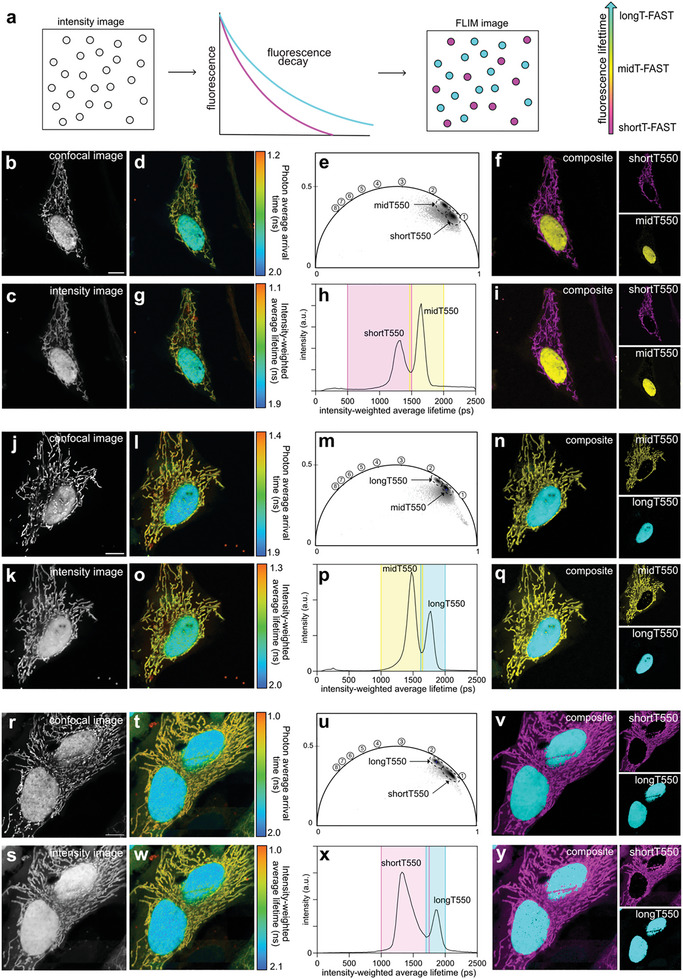
Multiplexed imaging of fluorescence lifetime‐modulating tags labeled with HBR‐2,5DM. a) Principle of lifetime‐based separation of two FAST:fluorogen assemblies within the same sample. b–y) Combinations of mitochondrial shortT550 and nuclear midT550 b–i), mitochondrial midT550 and nuclear longT550 j–q) and mitochondrial shortT550 and nuclear longT550 r–y). For each pair, the variants were expressed in HeLa cells. For each pair, are shown b,j,r) the confocal image, c,k,s) the intensity image, d,l,t) the photon average arrival time image and g,o,w) the intensity‐weighted average lifetime coded image (fit with biexponential model). e,m,u) Phasor plot analysis. Each cluster corresponds to a variant (time in ns is shown on the universal circle). The clusters used for separation are circled. f,n,v) Composite image resulting from the clustering shown on the phasor plot (shortT‐FAST is in magenta, midT‐FAST in yellow, longT‐FAST in cyan). h,p,x) Intensity‐weighted average lifetime distribution of the sample. Each peak corresponds to a variant. In color, are shown the two windows of lifetimes used for separation. i,q,y) Composite image resulting from the fitting‐based separation (shortT‐FAST is in magenta, midT‐FAST in yellow, longT‐FAST in cyan). Representative results of >16 cells from at least three biological replicates. Scale bars, 10 µm. b,j,r) Excitation 488 nm/detection window 500–600 nm. e,m,u) Phasor plot images were inverted using ezReverse, an online app for inverting background (https://github.com/Morwey/ezreverse).^[^ ^20^ ^]^ See Annex Figure 1 for brightness quantification.

**Figure 3:**
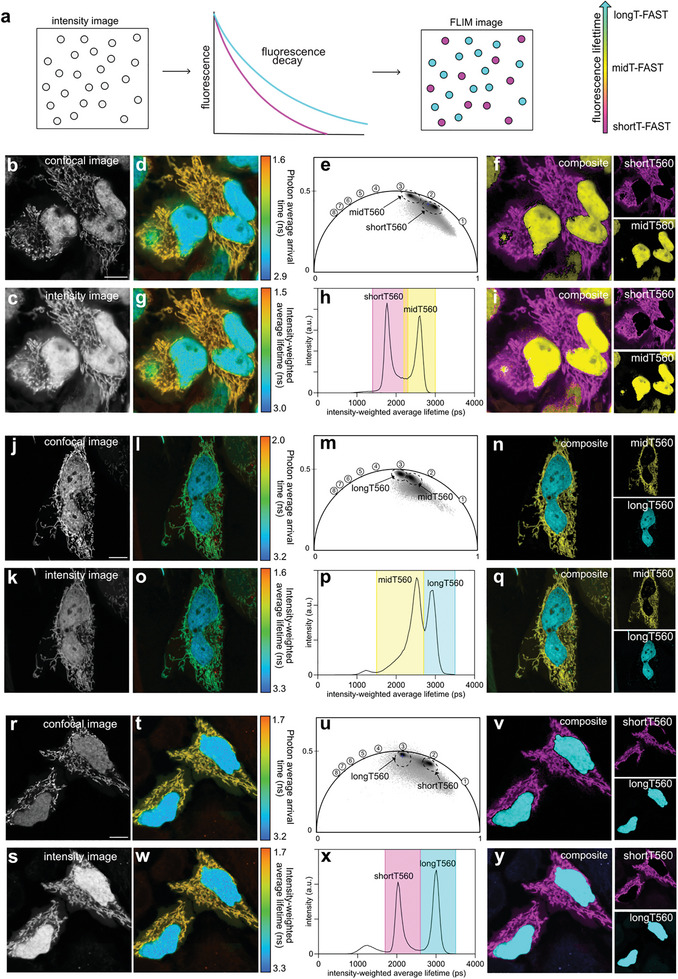
Multiplexed imaging of fluorescence lifetime‐modulating tags labeled with HBR‐3,5DM. a) Principle of lifetime‐based separation of two FAST:fluorogen assemblies within the same sample. b–y) Combinations of mitochondrial shortT560 and nuclear midT560 b–i), mitochondrial midT560 and nuclear longT560 j–q) and mitochondrial shortT560 and nuclear longT560 r–y). For each pair, the variants were expressed in HeLa cells. For each pair, are shown b,j,r) the confocal image, c,k,s) the intensity image, d,l,t) the photon average arrival time image and g,o,w) the intensity‐weighted average lifetime coded image (fit with biexponential model). e,m,u) Phasor plot analysis. Each cluster corresponds to a variant (time in ns is shown on the universal circle). The clusters used for separation are circled. f,n,v) Composite image resulting from the clustering shown on the phasor plot (shortT‐FAST is in magenta, midT‐FAST in yellow, longT‐FAST in cyan). h,p,x) Intensity‐weighted average lifetime distribution of the sample. Each peak corresponds to a variant. In color, are shown the two windows of lifetimes used for separation. i,q,y) Composite image resulting from the fitting‐based separation (shortT‐FAST is in magenta, midT‐FAST in yellow, longT‐FAST in cyan). Representative results of over >13 cells from at least three biological replicates. Scale bars, 10 µm. b) Excitation 488 nm/detection window 517–600 nm. j,r) Excitation 488 / detection window 517–570 nm. e,m,u) Phasor plot images were inverted using ezReverse, an online app for inverting background (https://github.com/Morwey/ezreverse).^[^ ^20^ ^]^ See Annex Figure 2 for brightness quantification.

To demonstrate the possibility of performing fluorescence lifetime multiplexing, we first tested pairwise combinations of shortT‐FAST, midT‐FAST, and longT‐FAST labeled with either HBR‐2,5DM (**Figure** **2**) or HBR‐3,5DM (**Figure** **3**). To test each pair, we co‐expressed the two variants in HeLa cells in two different cellular localizations, the nucleus and mitochondria, in order to assign one localization to one variant. Nuclear localization was obtained through the fusion of the histone H2B, while expression in the matrix of mitochondria was obtained through fusion to the mitochondrial targeting sequence (mito) from the subunit VIII of human cytochrome C oxidase. While the lifetime signature of an emissive species does not depend on its concentration, attention must be given to fluorescence levels to achieve good photon counting, in particular, when multiplexing reporters with different cellular brightness.^[^ ^7^ ^]^ We thus optimized, for each pair, the expression of the two variants to achieve similar fluorescence signals for better photon counting (see Experimental Section for details and Annex Figures in Supporting Information for systematic quantification of the brightness of the images shown in the paper). Various techniques can be used to analyze the FLIM data and separate the contributions of two fluorophores. For each pair of reporters, we compared the photon average arrival time image with the fluorescence decay fitting method and the fit‐free phasor approach for separation of the individual variants. Each separation technique bears different advantages and drawbacks: while the fitting approach is widely used to report values of fluorescence lifetime and characterize a given fluorescent reporter, the phasor approach does not require any assumption on the mono, bi, or triexponential decay behavior of the reporter's lifetime and gives a visual representation of the different fluorescent populations present in a given sample. In our case, while the different fluorescent assemblies were undistinguishable in the intensity‐only image, we achieved proper discrimination of each variant of a given pair no matter the analysis method (Figures 2 and 3), in agreement with their differences in lifetime. For each pair of variants, mitochondria and nucleus displayed different photon average arrival times, in agreement with the presence of two different variants in these two locations (Figure 2d,l,t, and Figure 3d,l,t). The phasor representation was used to visualize these two populations, which, for each pair, appear as separate clusters (Figure 2e,m,u and Figure 3e,m,u). Segmentation based on the position of these clusters allowed to separate the fluorescence lifetime‐modulating tags expressed in the mitochondria and the nucleus (Figure 2f,n,v and Figure 3f,n,v). Separate lifetime signatures were also observed on the intensity‐weighted average lifetime image obtained through fitting of the fluorescence decays with a biexponential model (Figure 2h,p,x and Figure 3h,p,x). For all assemblies, the intensity‐weighted average lifetime distribution shows two separate bands, allowing the definition of two temporal windows for lifetime‐based separation of the two variants (Figure 2i,q,y and Figure 3i,q,y). The average lifetime difference remained close to the lifetime difference measured when the variants were expressed alone as H2B fusions in HeLa cells (Figure S8, Supporting Information). Note that, with HBR‐3,5DM, the discrimination of midT560 and longT560 was more challenging because of the larger overlap of the two individual lifetime distributions in this context. However, as observed during our initial characterization, shortT560 and longT560 displayed the largest lifetime difference (ΔT_i_ > 1 ns), which makes this pair the best for FLIM multiplexing of two targets.

**Figure 4:**
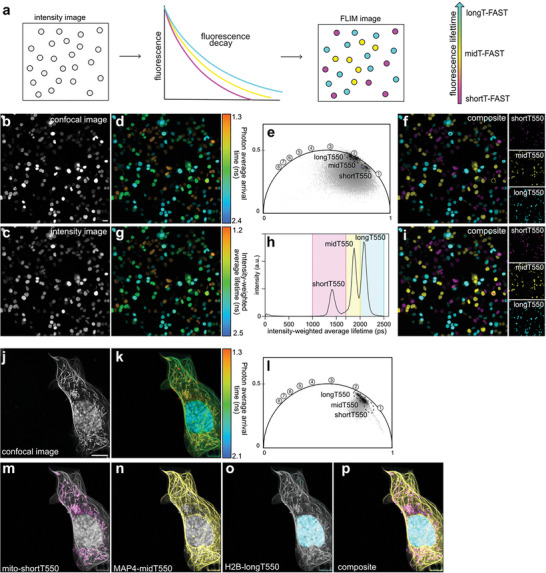
Multiplexed imaging with three fluorescence lifetime‐modulating tags. a) Principle of lifetime‐based separation of three FAST:fluorogen assemblies within the same sample. b–i) Populations of HEK293T cells expressing either H2B−shortT‐FAST or H2B−midT‐FAST or H2B−longT‐FAST were mixed together, labeled with 10 µM HBR‐2,5DM, and imaged by confocal microscopy and FLIM. Are shown b) the confocal image (Excitation 488 nm/detection window 517–600 nm), c) the intensity image, d) the photon average arrival time image and g) the intensity‐weighted average lifetime coded image (fit with biexponential model). e) Phasor plot analysis. Each cluster corresponds to a variant (time in ns is shown on the universal circle). The clusters used for separation are circled. f) Composite image resulting from the clustering shown on the phasor plot (shortT‐FAST is in magenta, midT‐FAST in yellow, longT‐FAST in cyan). h) Intensity‐weighted average lifetime distribution of the sample. In color, are shown the three windows of lifetimes used for separation. i) Composite image resulting from the fitting‐based separation (shortT‐FAST is in magenta, midT‐FAST in yellow, longT‐FAST in cyan). Scale bars, 20 µm. j–p) U2OS cell expressing H2B−longT‐FAST, mito−shortT‐FAST, and MAP4−midT‐FAST and labeled with 10 µM HBR‐2,5DM were imaged by confocal microscopy and FLIM. Are shown j) the confocal image (Excitation 488 nm/detection window 517–600 nm), k) the photon average arrival time image. The cell shown in this image was manually masked to allow proper phasor analysis. l–p) The three variants were separated using the indicated clusters on the phasor representation l) and overlayed over the intensity image m–p) (shortT‐FAST is in magenta, midT‐FAST in yellow, longT‐FAST in cyan). Representative results of three independent experiments. Scale bars, 10 µm. e,l) Phasor plot images were inverted using ezReverse, an online app for inverting background (https://github.com/Morwey/ezreverse).^[^ ^20^ ^]^ See Annex Figure 3 for brightness quantification.

Based on the analysis of their fluorescence lifetimes, we reasoned that it should be possible to separate shortT550, midT550, and longT550 in the same sample. To demonstrate lifetime multiplexing of these three variants, we first co‐cultured populations of HEK293T mammalian cells expressing respectively H2B–shortT‐FAST, H2B–midT‐FAST, and H2B–longT‐FAST and imaged them in presence of HBR‐2,5DM by spectral confocal microscopy and FLIM (**Figure** **4**a). While spectral confocal microscopy did not allow separation of shortT550, midT550 and longT550 because of their identical spectral properties (Figure 4b,c), efficient discrimination was possible through FLIM, as demonstrated by the presence of three separate lifetime populations on the photon average arrival time image (Figure 4d) as well as three separate clusters on the phasor plot (Figure 4e), enabling the separation of the three variants (Figure 4f). When fitting the fluorescence decays with biexponential model (Figure 4 g), three peaks in the intensity‐weighted average lifetime distribution histogram were visible at T_i_ = 1.45, 1.89, and 2.10 ns respectively, that were attributed respectively to shortT550, midT550 and longT550 (Figure 4 h), allowing the separation of the three variants (Figure 4i).

To further evaluate our ability to separate shortT550, midT550 and longT550, we co‐expressed in U2OS shortT‐FAST, midT‐FAST, and longT‐FAST, respectively in fusion with mitochondrial targeting sequence (mito) from the subunit VIII of human cytochrome C oxidase (for expression in the mitochondria matrix), microtubule‐associated protein (MAP)4 (for targeting microtubules) and H2B (for expression in the nucleus), and labeled them with HBR‐2,5DM (Figure 4j,k). Phasor plot‐based identification of clusters allowed the separation of the three reporters and their visualization in the expected localization (Figure 4l–p). In this context, the phasor plot approach facilitated the separation of the three reporters using the positions of the individual clusters.

To further push fluorescence lifetime multiplexing in the green channel, we imaged shortT550, midT550, and longT550 together with the enhanced green fluorescent protein (EGFP) using the co‐culture experiment described above. Since the reported fluorescence lifetime of EGFP is longer than that of longT550 (T_i_ = 2.6 ns,  ΔT_i_ = 0.5 ns),^[^ ^5^ ^]^ we reasoned that separation of four variants in the green channel would be possible. To unmistakably distinguish EGFP from the FAST variants, we expressed it in the cytosol (Figure S9a,d, Supporting Information). The phasor plot of the corresponding sample displayed four separated clusters (Figure S9b,c, Supporting Information), and the longest lifetime corresponded to the cells with cytosolic fluorescence, in agreement with EGFP localization. The intensity‐weighted average lifetime distribution in fields of view comprising cells expressing the four different reporters displayed four separated peaks, with maxima at T_i_ = 1.26, 1.64, 1.82, and 2.08 ns, corresponding respectively to shortT550, midT550, longT550, and EGFP (Figure S9e,f, Supporting Information).

This set of experiments allowed us to demonstrate the possibility of achieving lifetime multiplexing of three different FAST variants with identical spectral properties in living cells.

### 2.3 Fluorescence Lifetime Multiplexing in Zebrafish Larvae

**Figure 5:**
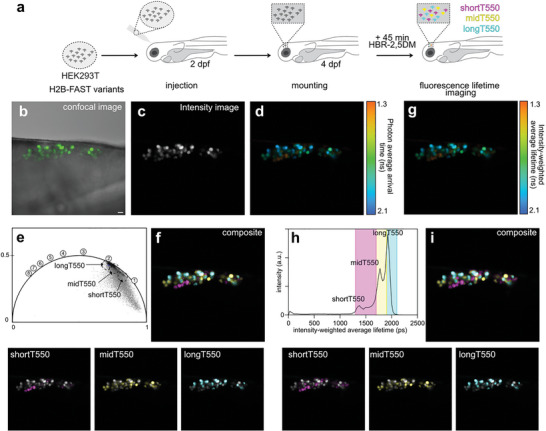
FLIM multiplexing in live zebrafish larvae. a) Three populations of mammalian HEK293T were transfected with plasmid encoding either H2B‐shortT‐FAST, H2B‐midT‐FAST, and H2B‐longT‐FAST. After 24 h, they were mixed together and they were injected near the developing brain of 2 dpf zebrafish larvae. Larvae were imaged at 4 dpf after 45 min incubation with HBR‐2,5DM. Are shown b) the confocal image (Excitation 488 nm/detection window 508–570 nm), c) the intensity image, d) the photon average arrival time image, and g) the intensity‐weighted average lifetime coded image (fit with biexponential model). e) Phasor plot analysis (time in ns is shown on the universal circle). The clusters used for separation are circled. Phasor plot image was inverted using ezReverse, an online app for inverting background (https://github.com/Morwey/ezreverse).^[^ ^20^ ^]^ f) Composite image resulting from the clustering shown on the phasor plot. Separate channels are overlayed on the intensity channel (shortT‐FAST is in magenta, midT‐FAST in yellow, and longT‐FAST in cyan). h) Intensity‐weighted average lifetime distribution of the sample. In color, are shown the three windows of lifetimes used for separation. i) Composite image resulting from the fitting‐based separation. Separate channels are overlayed on the intensity channel (shortT‐FAST is in magenta, midT‐FAST in yellow, and longT‐FAST in cyan). Scale bars, 20 µm. Representative results of three independent experiments. See Annex Figure 4 for brightness quantification.

Next, we demonstrated our ability to achieve fluorescence lifetime multiplexing of shortT‐FAST, midT‐FAST, and longT‐FAST in zebrafish larvae as model of multicellular organisms. Zebrafish is a common biological model to study a variety of biological processes such as development, inflammation, infection, cell proliferation, and metastasis.^[^ ^15^, ^16^ ^]^ In this context, cancer cells expressing fluorescent reporters can be injected in zebrafish embryos and larvae and their interaction with their environment as well as their proliferation can be assessed through fluorescence microscopy. To demonstrate fluorescence lifetime multiplexing of shortT550, midT550, and longT550 in a single spectral channel in zebrafish larvae, we injected a mix of three HEK293T cell populations expressing respectively H2B–shortT‐FAST, H2B–midT‐FAST, and H2B–longT‐FAST (**Figure** **5**a) in the developing brain. Two days later, larvae were imaged after treatment with HBR‐2,5DM. Confocal imaging of the larvae shows clusters of green‐emitting cells near the injection point (Figure 5b,c). The fluorescence lifetimes of each variant were also assessed independently through injection of the individual cell populations (Figure S10, Supporting Information) and were consistent with those measured previously in cultured mammalian cells (1.45 ns for shortT550, 1.70 ns for midT550 and 1.93 ns for longT550, Figure S10d,h,l, Supporting Information). Although some structures of the zebrafish larvae displayed some autofluorescence, their fluorescence lifetime was shorter than those of the FAST:fluorogen assembly, enabling to exclude autofluorescence by selecting only the fluorescence lifetime of the reporters. Fluorescence lifetime multiplexing allowed us to discriminate the three fluorescence lifetime‐modulating tags in the zebrafish larvae, as demonstrated by the presence of three fluorescence lifetime signatures distinguishable in the phasor plot (Figure 5e,f) as well as in the intensity‐weighted average lifetime distribution (Figure 5g–i). This set of experiments allowed us to demonstrate the possibility of achieving fluorescence lifetime multiplexing of three targets in a single spectral channel in a multicellular organism.

### 2.4 Fluorescence Lifetime Multiplexing Combined with Spectral Multiplexing

**Figure 6:**
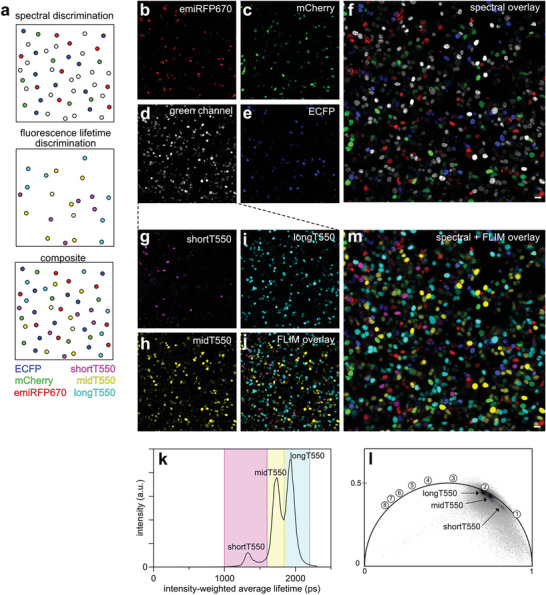
Spectral and lifetime multiplexing in live cells. a) Principle of combined spectral and lifetime‐based separation of six fluorescent reporters. Confocal micrographs of HEK293T cells expressing H2B‐emiRFP670 (Excitation 639 nm/detection window 650–757 nm) b), H2B‐mCherry (Excitation 561 nm/detection window 600–632 nm) c), H2B‐shortT‐FAST, H2B‐midT‐FAST, and H2B‐longT‐FAST (Excitation 488 nm/detection window 508–570 nm) d), and H2B‐ECFP (Excitation 445 nm/detection window 455–473 nm) e) in addition to spectral overlay f). Scale bars, 20 µm. FLIM was then used to discriminate reporters in the green channel based on their intensity‐weighted average lifetime distribution g–k). The phasor plot in the green channel is also shown l) (time in ns is shown on the universal circle). Phasor plot image was inverted using ezReverse, an online app for inverting background (https://github.com/Morwey/ezreverse).^[^ ^20^ ^]^ m) spectral and fluorescence lifetime overlay. Representative results of three independent experiments. Scale bar, 20 µm.

Finally, we further pushed multiplexing by combining fluorescence lifetime multiplexing with spectral multiplexing. We combined shortT550, midT550, and longT550 in the green channel, together with cyan, red, and near‐infrared (NIR) fluorescent reporters. For this purpose, we co‐cultured mammalian cells expressing respectively ECFP (cyan channel), shortT‐FAST, midT‐FAST, and longT‐FAST (green channel), mCherry (red channel), and emiRFP670 (NIR channel) and labeled the cells with HBR‐2,5DM (**Figure** **6**a). Confocal imaging allowed the spectral discrimination of ECFP, mCherry, and emiRFP670 in addition to a population of green‐emitting cells (Figure 6b–f) and we used FLIM to separate the green fluorescence emitting reporters. Similarly to previous multiplexing experiments, fitting approach (Figure 6g–k) and phasor plot (Figure 6l) allowed the identification of three lifetime signatures, in agreement with the different lifetimes of shortT550, midT550, and longT550. By combining spectral and lifetime imaging, we were able to image and distinguish up to six different fluorescent reporters (Figure 6m).

## 3 Discussion

In this work, we leveraged a collection of fluorescence‐activating and absorption‐shifting tag (FAST) variants with broad sequence diversity to identify fluorescence lifetime‐modulating tags allowing multiplexed imaging in a single spectral channel. We screened the fluorescence lifetimes of twelve different variants with three prototypical fluorogens forming green‐yellow fluorescent assemblies. Comprehensive characterization of greenFAST, oFAST, and TsiA‐FAST, renamed shortT‐FAST, midT‐FAST, and longT‐FAST, respectively, showed that these three variants formed fluorescent assemblies with HBR‐2,5DM and HBR‐3,5DM that display distinct fluorescence lifetime signatures, allowing their separation by FLIM although their spectral properties are identical. Our experiments showed that fluorescence lifetime is a robust parameter. Comparable lifetimes were found in the different cell lines we tested. However, we recommend to systematically characterize the fluorescence lifetime of shortT‐FAST, midT‐FAST, and longT‐FAST when using them in a new cellular system. In addition, as for any genetically encoded tag, we also recommend to systematically test that fusion to shortT‐FAST, midT‐FAST, and longT‐FAST does not affect the function of the protein of interest and is not cytotoxic.

Our study showed that the differences in fluorescence lifetime between shortT‐FAST, midT‐FAST, and longT‐FAST are not due to differences in their fluorescence quantum yields, but rather originate from different non‐radiative deexcitation behaviors. Our results suggest that the fluorescence lifetimes of such fluorogenic reporters can be tuned through engineering of the binding pocket of the fluorophore, opening interesting prospects for engineering variants with more substantial differences in lifetime: while the screening strategy previously used during the directed evolution process of FAST proteins focused more on selecting the tightest and brightest assemblies, an additional screening based on fluorescence lifetime could enable in the future the identification of variants displaying more significant differences in lifetime, and allow multiplexing of more targets. Among the three variants highlighted in this study, longT‐FAST (a.k.a TsiA‐FAST) was not obtained through directed evolution like the two others, but by exploring proteins with homology relationships. While this homology‐based protein engineering strategy allowed the generation of variants with sequence similarity of 70%–78%, exploring the use of homologs with lower similarity may allow the generation of novel FAST variants with different photophysical behaviors and thus different fluorescence lifetime signatures.

To demonstrate the possibility of achieving fluorescence lifetime multiplexing, we performed pairwise combinations of shortT‐FAST, midT‐FAST, and longT‐FAST with either HBR‐2,5DM or HBR‐3,5DM and showed that, for a given fluorogen, proper discrimination of each possible pair could be achieved. With HBR‐2,5DM, shortT550, midT550, and longT550 could be combined in the same experiment for fluorescence lifetime multiplexing of three targets in a single spectral channel as demonstrated in cultured mammalian cells. We leveraged this feature in zebrafish larvae through injection of a mixture of three mammalian cell populations, each expressing a different fluorescence lifetime‐modulating tag, and successfully distinguished them via their different lifetime signatures.

One interesting feature of fluorescence lifetime multiplexing is the possibility to distinguish variants with different lifetime signatures in one spectral channel, freeing up the other spectral channels for observing additional biological targets.^[^ ^17^, ^18^ ^]^ The fluorescence lifetime‐modulating tags identified in this study are green fluorescent. We demonstrated that spectral multiplexing and lifetime multiplexing could be combined to discriminate up to six different fluorescent reporters, opening prospects for monitoring as many different processes simultaneously.

The identification of fluorescence lifetime‐modulating tags opens new prospects for advanced multiplexed experiments. We demonstrated fluorescence lifetime multiplexed imaging of spatially separated tags, which enabled to easily assign one pixel to one species. Unmixing of multiple fluorescent species in the same pixel is also possible, in particular for monoexponentially decaying fluorophores.^[^ ^5^ ^]^ For probes exhibiting multiexponential decay such as those presented in this study, unmixing is a bit more challenging, however specific approaches to unmix the lifetimes of multiexponentially decaying fluorophores exist, opening exciting prospects for future applications.^[^ ^19^ ^]^ Beyond multiplexed imaging, fluorescence lifetime‐modulating tags could also be used for the design of lifetime responsive biosensors through coupling with recognition domains of specific analytes and biomolecules of interest. We believe fluorescence lifetime multiplexing using chemogenetic fluorescence lifetime‐modulating tags opens exciting prospects for biological imaging: combined with spectral multiplexing, it opens countless opportunities for observing several biological targets simultaneously.

## Conflict of Interest

The authors declare the following competing financial interest: A.G. is co‐founder and holds equity in Twinkle Bioscience/The Twinkle Factory, a company commercializing the FAST technology. The other authors declare no competing interests.

## Author Contributions

L.E.H and A.G. designed the overall project and wrote the paper with the help of the other authors; L.E.H, F.L., C.R., M.V., S.V., and A.G. designed the experiments; L.E.H, F.L., M.A., H.B., C.R., M.V., and S.V. performed the experiments; L.E.H, F.L., C.R., M.V., S.V., and A.G. analyzed the experiments.

## Supporting information

Supporting Information

## Data Availability

The data that support the findings of this study are available from the corresponding author upon reasonable request.
