# Supplementary material for: Multiplexed In Vivo Imaging with Fluorescence Lifetime‐Modulating Tags
Source: Adv Sci (Weinh). 2024 Jun 20;11(32):2404354. doi: 10.1002/advs.202404354 (PMC11347991; doi:10.1002/advs.202404354)
Supplement: Supplementary file 1 — Supporting Information [file ADVS-11-2404354-s001.pdf]

ADVANCED SCIENCE
Open Access

Supporting Information

for *Adv. Sci.*, DOI 10.1002/advs.202404354

Multiplexed In Vivo Imaging with Fluorescence Lifetime-Modulating Tags

Lina El Hajji, France Lam, Maria Avtodeeva, Hela Benaissa, Christine Rampon, Michel Volovitch, Sophie Vriz and Arnaud Gautier**

Supporting information

Multiplexed in vivo imaging with fluorescence lifetime-modulating tags

Lina El Hajji[1,*], France Lam[2], Maria Avtodeeva[1], Hela Benaissa[1], Christine Rampon[1,3], Michel Volovitch[1], Sophie Vriz[1,3] & Arnaud Gautier[1,4,*]

[1] Sorbonne Université, École Normale Supérieure, Université PSL, CNRS, Laboratoire des Biomolécules, LBM, 75005 Paris, France

[2] Sorbonne Université, CNRS, Institut de Biologie Paris Seine, plateforme imagerie photonique I2PS (FR3631), 75005 Paris, France

[3] Université Paris Cité, 75006 Paris, France

[4] Institut Universitaire de France

* Correspondence should be addressed to: lina.el_hajji@sorbonne-universite.fr and arnaud.gautier@sorbonne-universite.fr

This PDF file includes:

Text S1

Figures S1-10

Tables S1-3

Experimental section

Annex Figures 1-5

Supplementary references

Text S1. The FAST family

The Fluorescence-activating and absorption-shifting tag (FAST) toolbox is a family of small chemogenetic reporters evolved from the 14-kDa photoactive yellow protein (PYP) found in the phototactic bacterium *Halorhodospira halophila*. Prototypical FAST binds hydroxybenzylidene rhodanine (HBR) derivatives[1,2] and stabilizes their fluorescent state. When they are free in solution, these so-called fluorogens dissipate energy through non-radiative de-excitation pathways. However, within the cavity of FAST, they adopt a quasi-planar conformation and the phenolate form of the fluorogens is stabilized through hydrogen bonding with residue E46, a key residue for the modulation of spectral properties of PYP.[3,4]

Since the development of prototypical FAST, the FAST family of reporters has been fairly extended through alteration of the structure of the fluorogens, as well as engineering of adequate cognate protein tags. Molecular engineering of the phenol ring of HBR scaffold allowed the design of new fluorogens displaying green to red emission in assembly with FAST, allowing to have different fluorescence emission properties with the same protein tag using either HBR-2,5DM, HBR-3,5DM and HBR-3,5DOM.[2] Rational design allowed the development of an enhanced version of FAST named iFAST, characterized by superior properties with HMBR.[5] Directed evolution later allowed the design of two orthogonal tags, greenFAST and redFAST, binding preferably HMBR and HBR-3,5DOM respectively.[6] The characterization of the fluorescence lifetime of greenFAST:HMBR assembly highlighted an interesting behaviour, as its fluorescence lifetime was shown to be shorter than that of iFAST:HMBR, although the two assemblies have similar fluorescence quantum yields (FQY). More recently, the FAST family was extended with three new reporters: pFAST, oFAST and tFAST, obtained through a concerted strategy of directed evolution and molecular engineering of the chromophore.[7] pFAST has the advantageous feature of being a promiscuous tag, able to bind and activate the fluorescence of different fluorogen families, enabling fluorescent assemblies spanning the visible spectrum.

While the previously cited FAST variants were engineered through rational design and directed evolution, a novel strategy based on protein homology allowed recently the development of six new FAST reporters, HboL-FAST, HspG-FAST, RspA-FAST, Ilo-FAST, TsiA-FAST and Rsa-FAST,[8] engineered from six homologs of PYP from *Halomonas boliviensis* LC1 (HboL), *Halomonas* sp. GFAJ-1 (HspG), *Rheinheimera* sp. A13L (RspA), *Idiomarina loihiensis* (Ilo), *Thiorhodospira sibirica* ATCC 700588 (TsiA) and *Rhodothalassium salexigens* (Rsa), displaying 70-78% sequence similarity with the original FAST sequence.

These FAST mutants constitute an interesting platform for the identification of variants which can be multiplexed based on their lifetime properties.

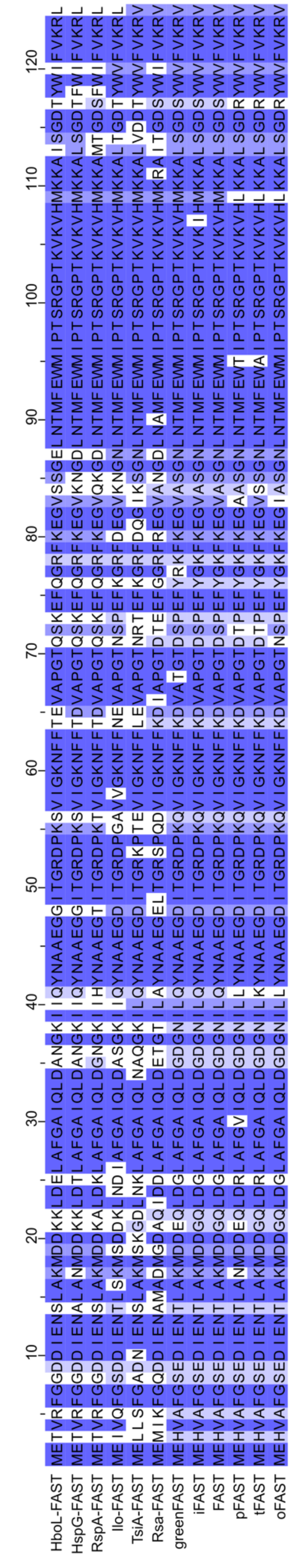

Figure S1. Alignment of the FAST variants used in this study.

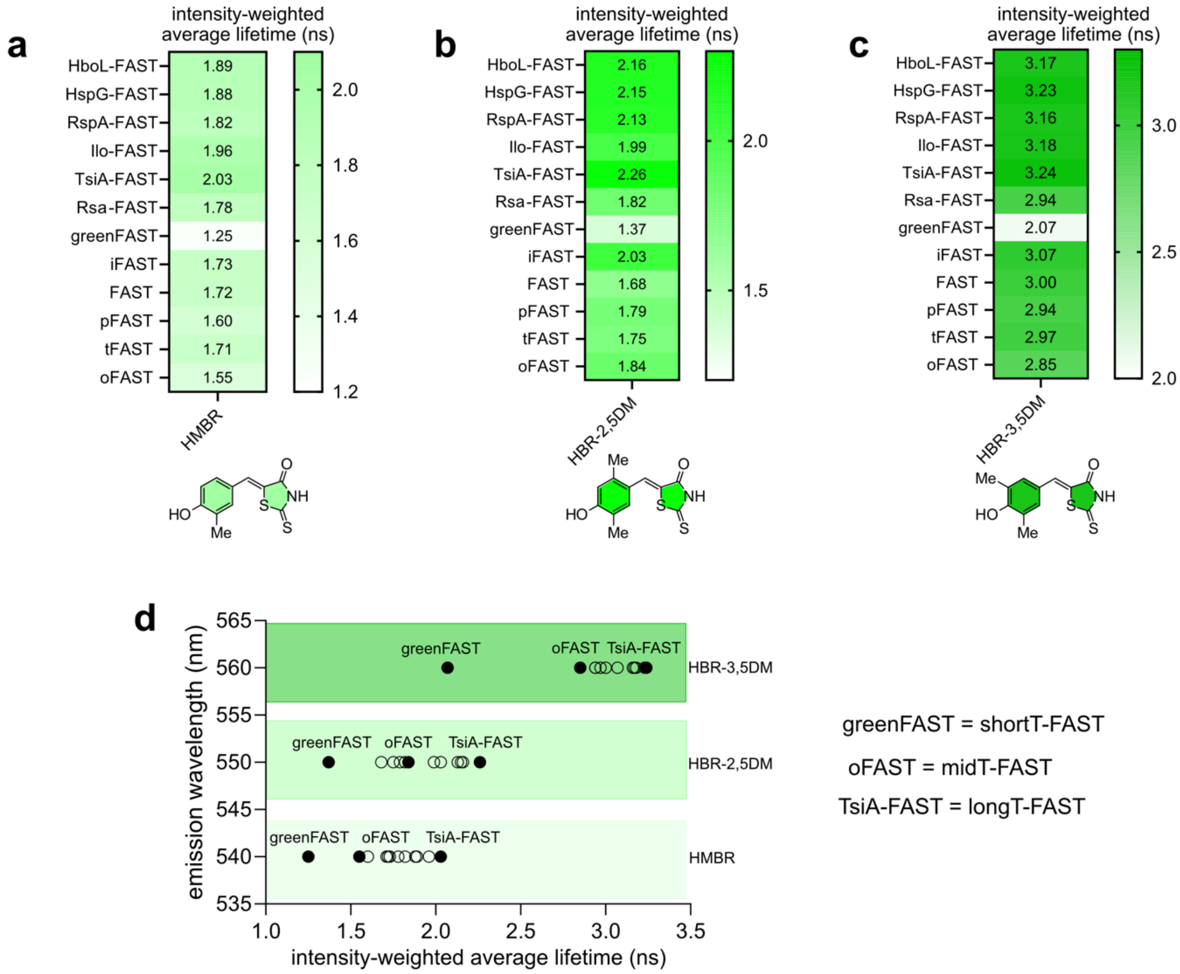

Figure S2. Fluorescence lifetime screening of FAST:fluorogen pairs. Mean (n = 8-16 cells) of intensity-weighted average lifetimes of FAST variants with (**a**) HMBR, (**b**) HBR-2,5DM and (**c**) HBR-3,5DM as measured in HEK293T cells. (**d**) Emission wavelengths of the FAST:fluorogen assemblies against their intensity-weighted average lifetimes. The data corresponding to greenFAST, oFAST and TsiA-FAST are shown as black full circles.

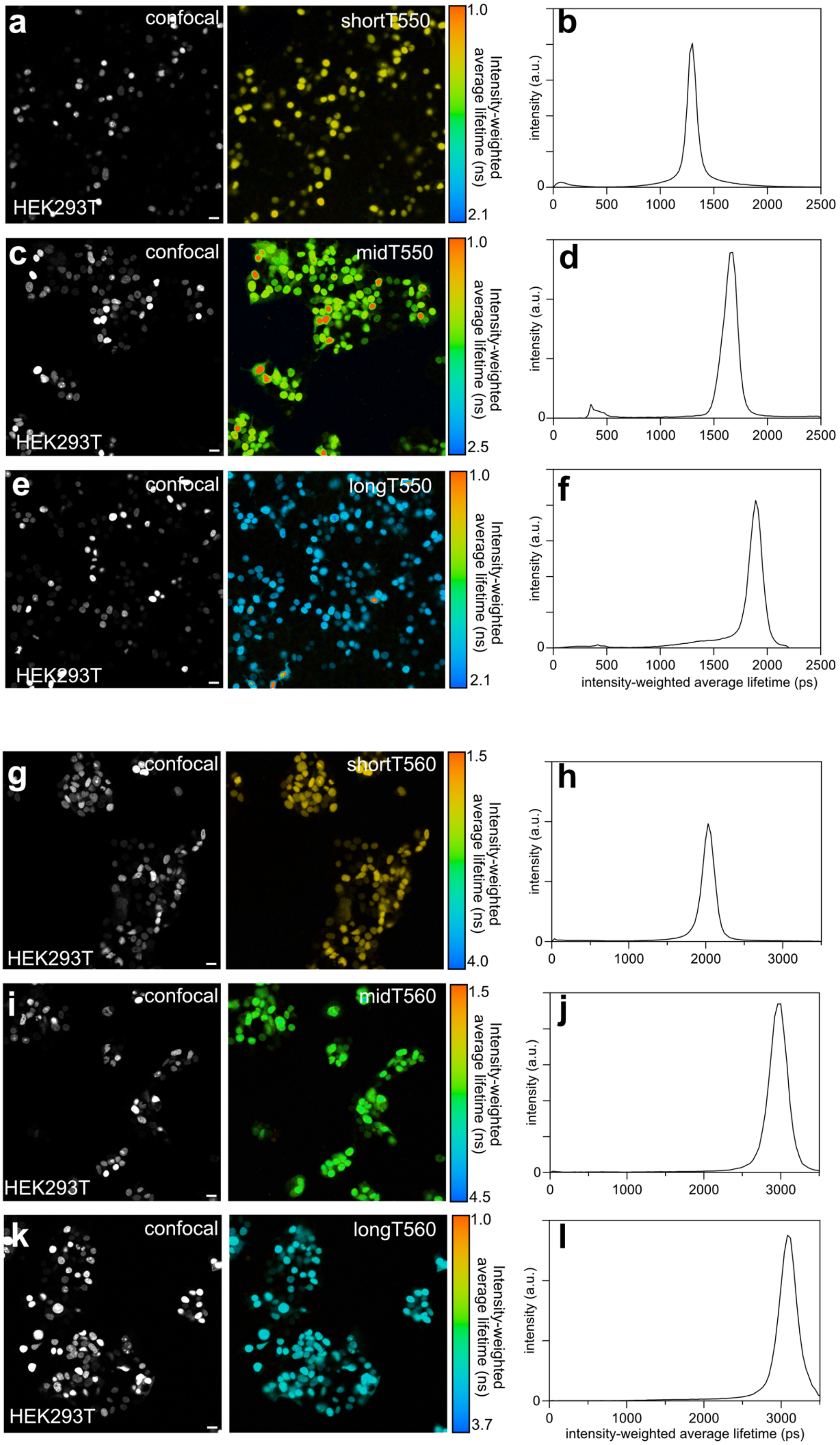
a
confocal
HEK293T
shortT550
1.0
2.1
Intensity-weighted average lifetime (ns)
b
intensity (a.u.)
0
0 500 1000 1500 2000 2500
c
confocal
HEK293T
midT550
1.0
2.5
Intensity-weighted average lifetime (ns)
d
intensity (a.u.)
0
0 500 1000 1500 2000 2500
e
confocal
HEK293T
longT550
1.0
2.1
Intensity-weighted average lifetime (ns)
f
intensity (a.u.)
0
0 500 1000 1500 2000 2500
intensity-weighted average lifetime (ps)
g
confocal
HEK293T
shortT560
1.5
4.0
Intensity-weighted average lifetime (ns)
h
intensity (a.u.)
0
0 1000 2000 3000
i
confocal
HEK293T
midT560
1.5
4.5
Intensity-weighted average lifetime (ns)
j
intensity (a.u.)
0
0 1000 2000 3000
k
confocal
HEK293T
longT560
1.0
3.7
Intensity-weighted average lifetime (ns)
l
intensity (a.u.)
0
0 1000 2000 3000
intensity-weighted average lifetime (ps)

Figure S3. Fluorescence lifetime characterization of shortT-FAST, midT-FAST and longT-FAST with HBR-2,5DM and HBR-3,5DM in HEK293T cells. Confocal micrographs, equivalent intensity-weighted average lifetime images and intensity-weighted average lifetime histogram (fit with biexponential model) of HEK293T cells expressing respectively H2B-shortT-FAST (**a,b,g,h**), H2B-midT-FAST (**c,d,i,j**) and H2B-longT-FAST (**e,f,k,l**) labeled with 10 μM HBR-2,5DM (**a-f**) or 10 μM HBR-3,5DM (**g-l**). Representative micrographs and distribution of at least five fields of view from two independent experiments. Scale bars, 20 μm. (**a,c,e,g,i,k**) Excitation wavelength 488 nm / Detection window 517-600 nm. See also Figure 1b and 1f for full quantification.

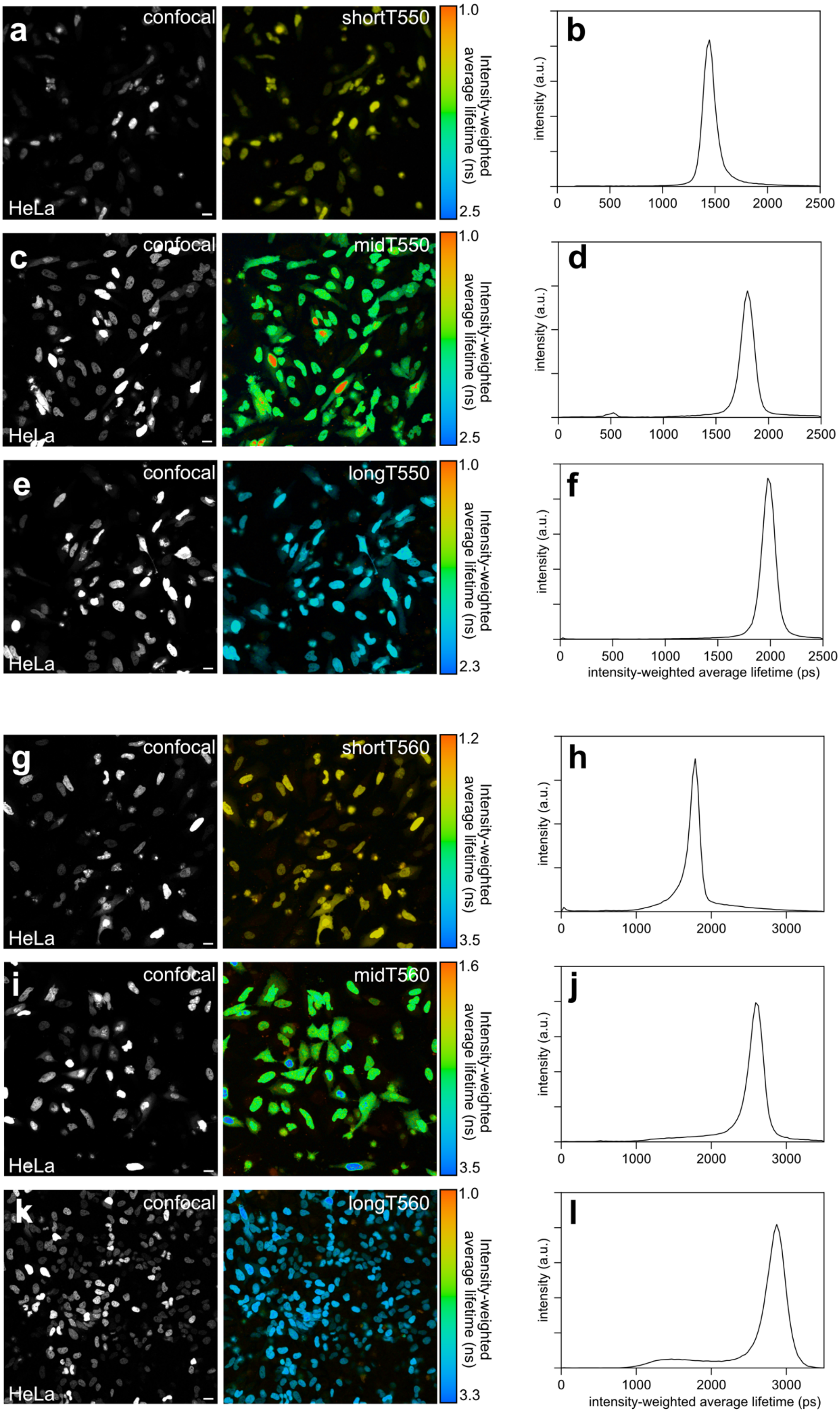
a
confocal
HeLa
shortT550
1.0
2.5
Intensity-weighted average lifetime (ns)
b
intensity (a.u.)
0 500 1000 1500 2000 2500
c
confocal
HeLa
midT550
1.0
2.5
Intensity-weighted average lifetime (ns)
d
intensity (a.u.)
0 500 1000 1500 2000 2500
e
confocal
HeLa
longT550
1.0
2.3
Intensity-weighted average lifetime (ns)
f
intensity (a.u.)
0 500 1000 1500 2000 2500
intensity-weighted average lifetime (ps)
g
confocal
HeLa
shortT560
1.2
3.5
Intensity-weighted average lifetime (ns)
h
intensity (a.u.)
0 1000 2000 3000
i
confocal
HeLa
midT560
1.6
3.5
Intensity-weighted average lifetime (ns)
j
intensity (a.u.)
0 1000 2000 3000
k
confocal
HeLa
longT560
1.0
3.3
Intensity-weighted average lifetime (ns)
l
intensity (a.u.)
0 1000 2000 3000
intensity-weighted average lifetime (ps)

Figure S4. Fluorescence lifetime characterization of shortT-FAST, midT-FAST and longT-FAST with HBR-2,5DM and HBR-3,5DM in HeLa cells. Confocal micrographs, equivalent intensity-weighted average lifetime images and intensity-weighted average lifetime histogram (fit with biexponential model) of HeLa cells expressing respectively H2B-shortT-FAST (**a**,**b**,**g**,**h**), H2B-midT-FAST (**c**,**d**,**i**,**j**) and H2B-longT-FAST (**e**,**f**,**k**,**l**) labeled with 10 μM HBR-2,5DM (**a**-**f**) or 10 μM HBR-3,5DM (**g**-**l**). Representative micrographs and distribution of at least six fields of view from two independent experiments. Scale bars, 20 μm. (**a**,**c**,**e**,**g**,**i**,**k**) Excitation 488 nm / detection window 517-600 nm. See also Figure 1c and 1g for full quantification.

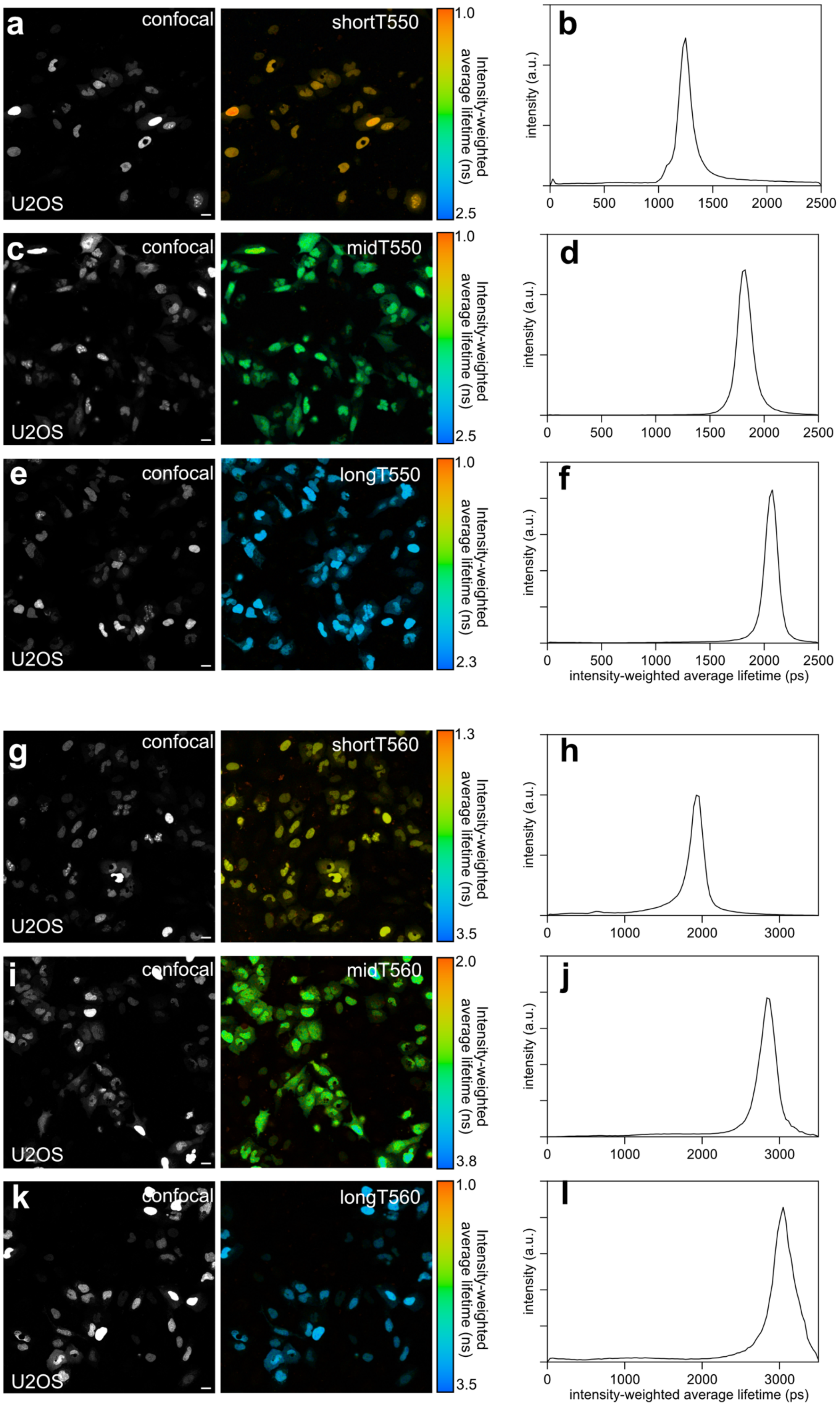
a
confocal
U2OS
shortT550
1.0
2.5
Intensity-weighted average lifetime (ns)
b
intensity (a.u.)
0
500
1000
1500
2000
2500
c
confocal
U2OS
midT550
1.0
2.5
Intensity-weighted average lifetime (ns)
d
intensity (a.u.)
e
confocal
U2OS
longT550
1.0
2.3
Intensity-weighted average lifetime (ns)
f
intensity (a.u.)
intensity-weighted average lifetime (ps)
g
confocal
U2OS
shortT560
1.3
3.5
Intensity-weighted average lifetime (ns)
h
intensity (a.u.)
0
1000
2000
3000
i
confocal
U2OS
midT560
2.0
3.8
Intensity-weighted average lifetime (ns)
j
intensity (a.u.)
k
confocal
U2OS
longT560
1.0
3.5
Intensity-weighted average lifetime (ns)
l
intensity (a.u.)
intensity-weighted average lifetime (ps)

Figure S5. Fluorescence lifetime characterization of shortT-FAST, midT-FAST and longT-FAST with HBR-2,5DM and HBR-3,5DM in U2OS cells. Confocal micrographs, equivalent intensity-weighted average lifetime images and intensity-weighted average lifetime histogram (fit with biexponential model) of U2OS cells expressing respectively H2B-shortT-FAST (**a,b,g,h**), H2B-midT-FAST (**c,d,I,j**) and H2B-longT-FAST (**e,f,k,l**) labeled with 10 µM HBR-2,5DM (**a-f**) or 10 µM HBR-3,5DM (**g-l**). Representative micrographs and distribution of at least six fields of view from two independent experiments. Scale bars, 20 µm. (**a,c,e,g,i,k**) Excitation 488 nm / detection window 517-600 nm. See also Figure 1d and 1h for full quantification.

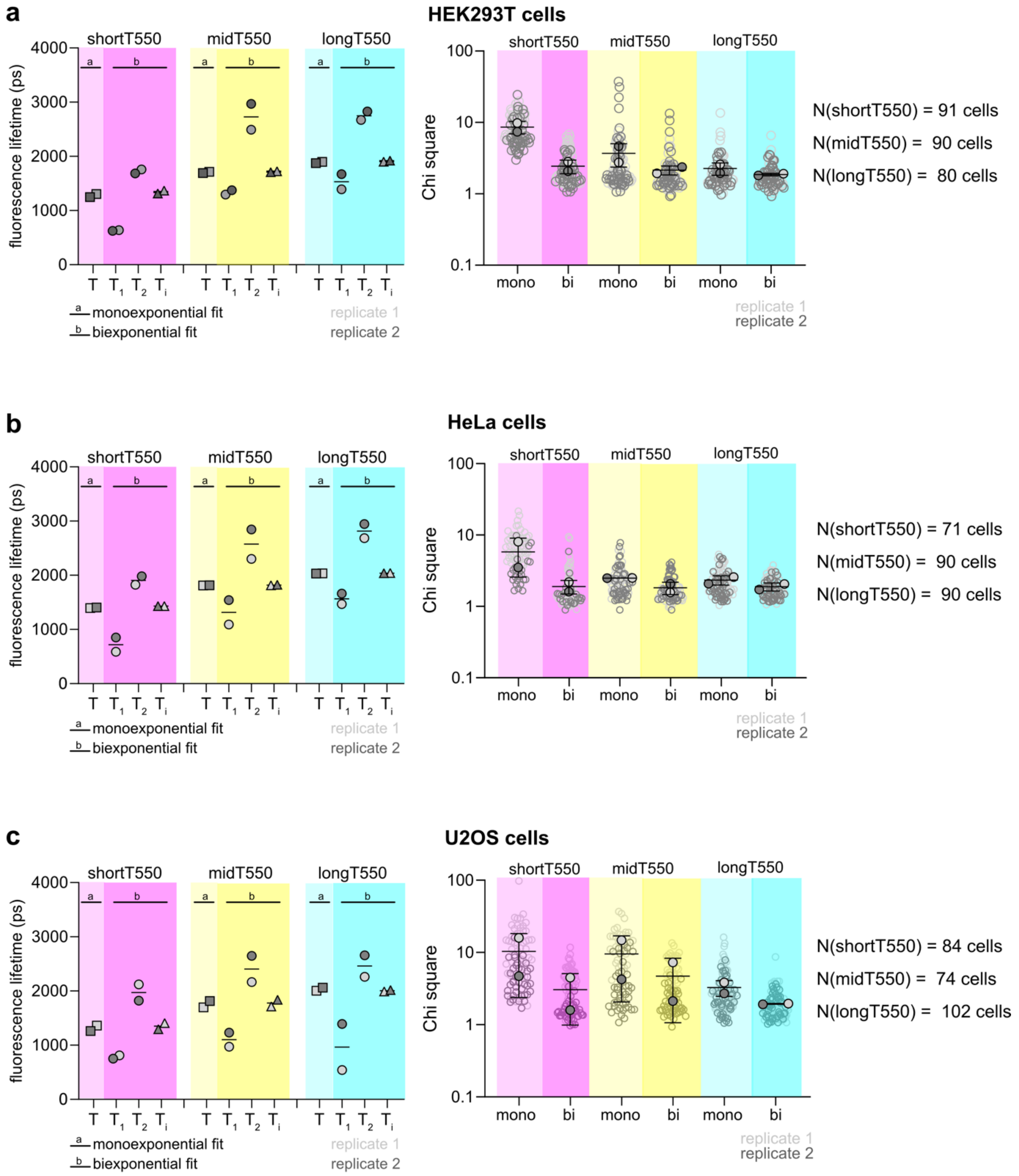

Figure S6. Comparison of the fitting models for the fluorescence lifetime analysis of shortT550, midT550 and longT550 in different cell lines. Means of fluorescence lifetime T of shortT550, midT550 and longT550 extracted from fitting with monoexponential model, as well as of the two fluorescence lifetime components T_1 and T_2 and intensity-weighted average fluorescence lifetime T_i from fitting with biexponential model in (**a**) HEK293T cells, (**b**) HeLa cells and (**c**) U2OS cells. N cells from two biological replicates were analyzed (the number N of analyzed cells is indicated on the right). The black lines represent the means of the two biological replicates. Each dot corresponds to the mean of each biological replicate (the lifetimes of individual cells are not shown). On the right is shown the distribution of goodness of fit (reduced χ^2 or chi square) of monoexponential and biexponential models. Each cell is color-coded according to the biological replicate it came from. The solid circles correspond to

the mean of each biological replicate. The black line represents the mean ± SD of the two biological replicates.

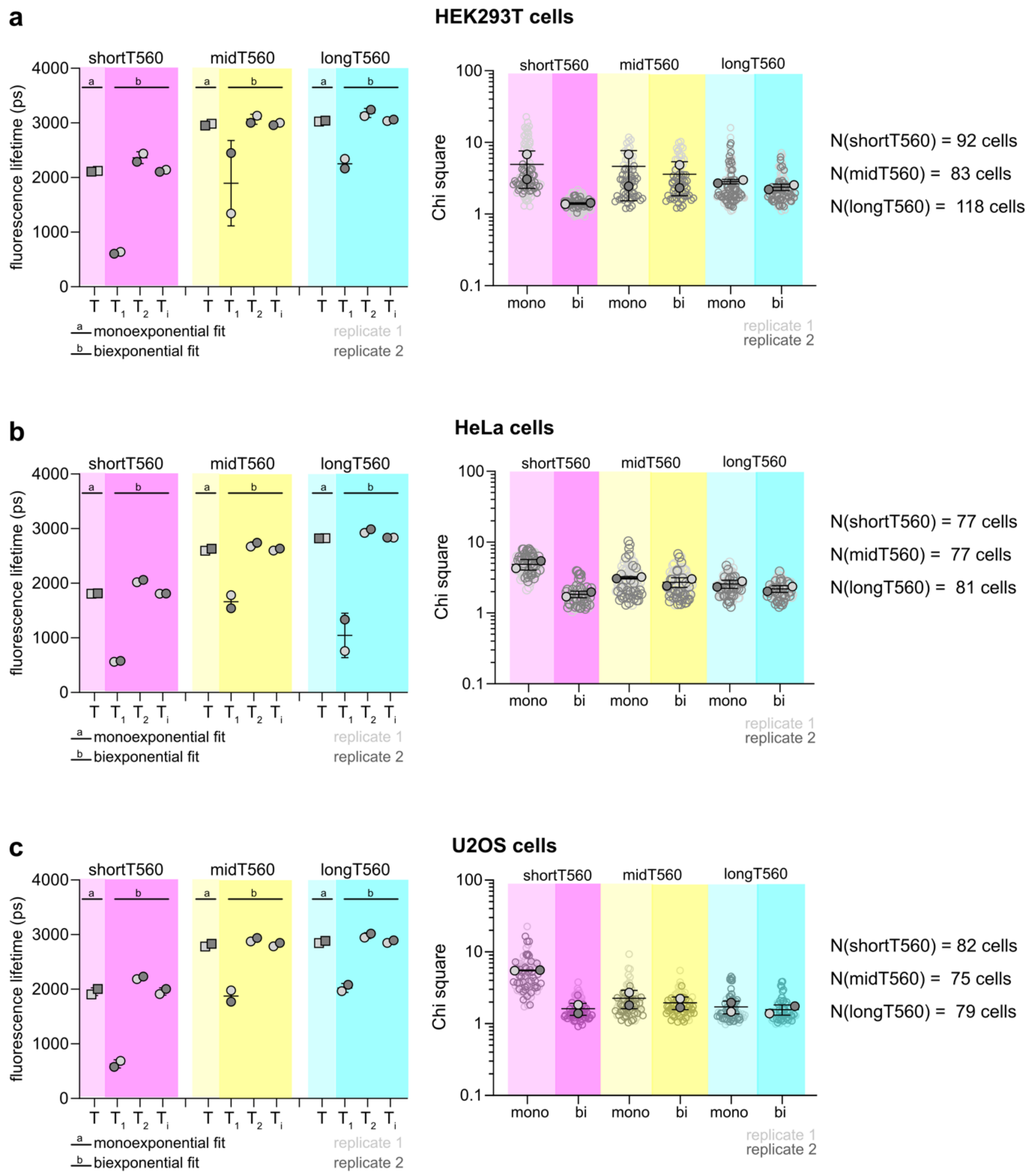

Figure S7. Comparison of the fitting models for the fluorescence lifetime analysis of shortT560, midT560 and longT560 in different cell lines. Means of fluorescence lifetime T of shortT560, midT560 and longT560 extracted from fitting with monoexponential model, as well as of the two fluorescence lifetime components T_1 and T_2 and intensity-weighted average fluorescence lifetime T_i from biexponential model in (**a**) HEK293T cells, (**b**) HeLa cells and (**c**) U2OS cells. N cells from two biological replicates were analyzed (the number of cells N is indicated on the right). The black lines represent the means of the two biological replicates. Each dot corresponds to the mean of each biological replicate (the lifetimes of individual cells are not shown). On the right is shown the distribution of goodness of fit (reduced χ^2 or chi square) of monoexponential and biexponential models. Each cell is color-coded according to

the biological replicate it came from. The solid circles correspond to the mean of each biological replicate. The black line represents the mean ± SD of the two biological replicates.

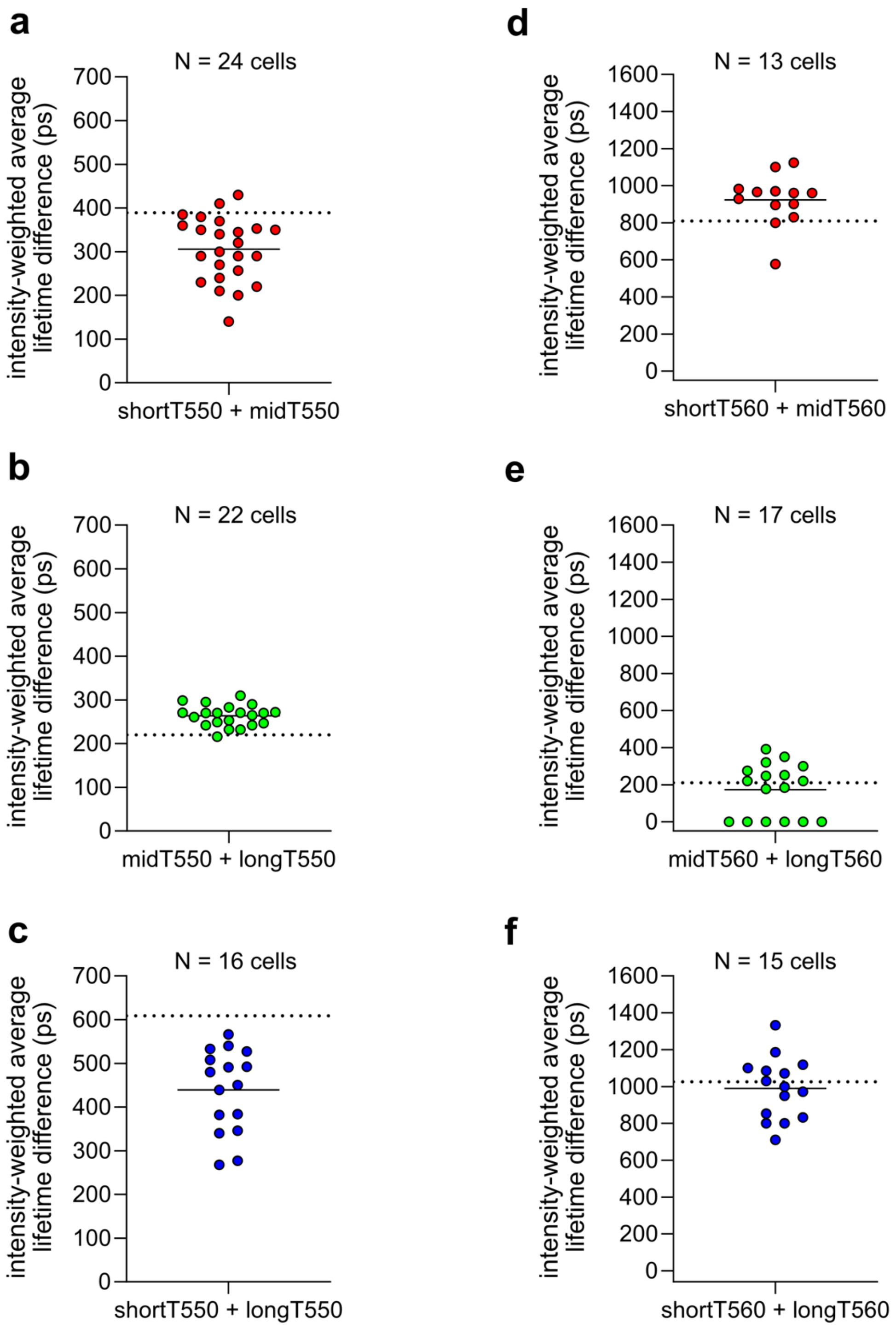

Figure S8. Pairwise intensity-weighted lifetime differences of shortT-FAST, midT-FAST and longT-FAST. Each dot corresponds to the differences in intensity-weighted between mito-shortT550 and H2B-midT550 (**a**), mito-midT550 and H2B-longT550 (**b**), mito-shortT550 and H2B-longT550 (**c**), mito-shortT560 and H2B-midT560 (**d**), mito-midT560 and H2B-longT560 (**e**) and mito-shortT560 and H2B-longT560 (**f**). The dotted line indicates the intensity-weighted average lifetime difference between H2B-FAST variants determined in HeLa cells during the individual variants characterization (see also Figure 1). The number of cells N (from at least three biological replicates) is indicated for each set of experiments.

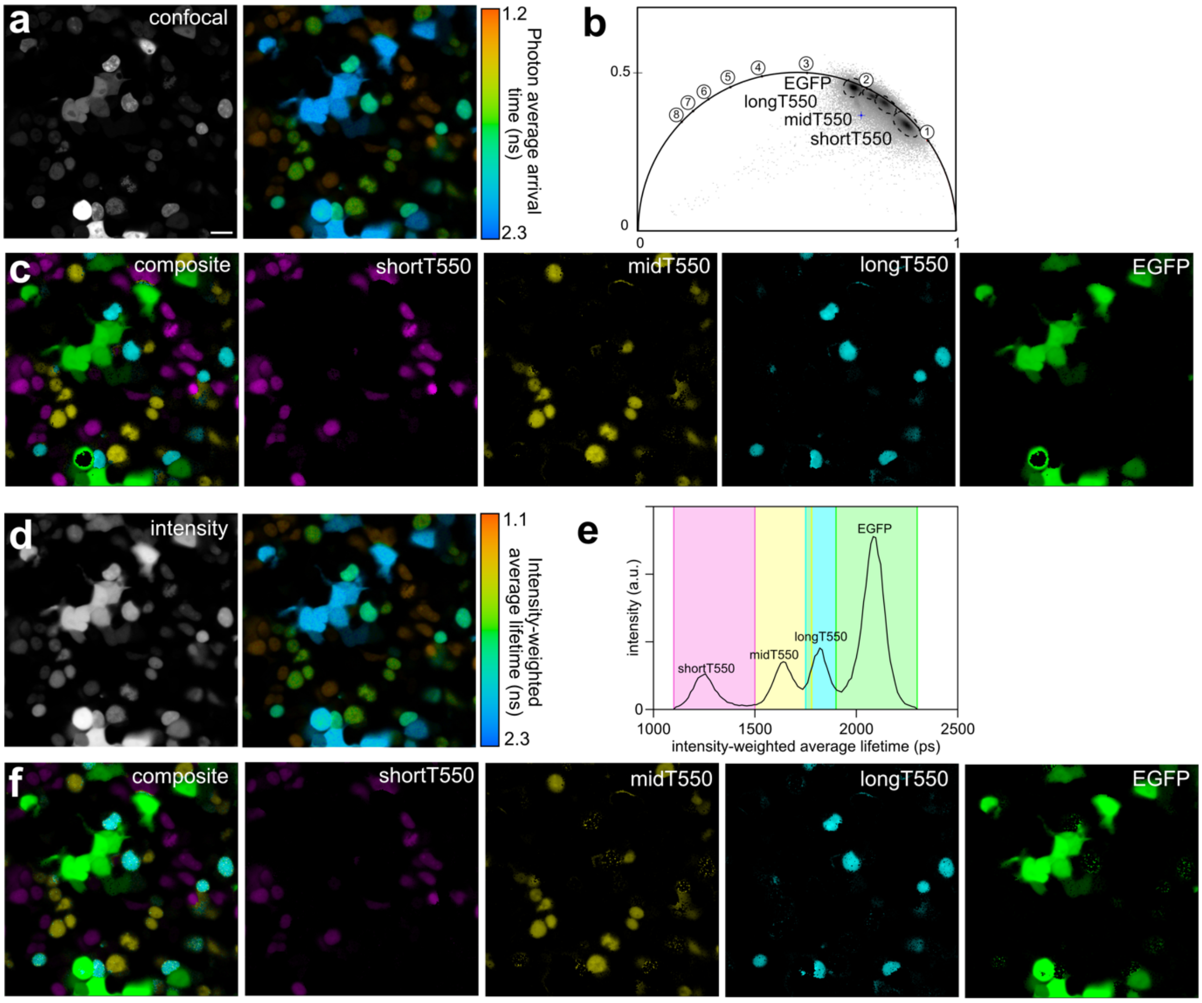

Figure S9. Fluorescence lifetime multiplexing in the green channel with shortT550, midT550, longT550 and EGFP. (**a**) Confocal micrographs and equivalent average arrival time of photons color-coded image of a mix of HEK293T cells expressing H2B–shortT-FAST, H2B–midT-FAST, H2B–longT-FAST and cytosolic EGFP and labeled with 10 µM HBR-2,5DM. Scale bars, 20 µm. Excitation 488 nm / detection window 508-570 nm. (**b**) Corresponding phasor representation showing four separable clusters assigned to shortT550, midT550, longT550 and EGFP (time in ns is shown on the universal circle). Phasor plot image was inverted using ezReverse, an online app for inverting background (https://github.com/Morwey/ezreverse).[9] (**c**) Phasor based separation of shortT550, midT550, longT550 and EGFP. Are shown the four individual isolated populations and a composite of all. (**d**) Intensity image micrographs and equivalent intensity-weighted average lifetime (biexponential fit) of a mix of HEK293T cells expressing H2B–shortT-FAST, H2B–midT-FAST, H2B–longT-FAST and cytosolic EGFP and labeled with 10 µM HBR-2,5DM. (**e**) Corresponding intensity-weighted average lifetime histogram showing four separated peaks, assigned to shortT550, midT550, longT550 and EGFP. (**f**) Intensity-weighted based separation of shortT550, midT550, longT550 and EGFP. Are shown the four individual isolated populations and a composite of all. Representative results of six fields of view from two biological replicates.

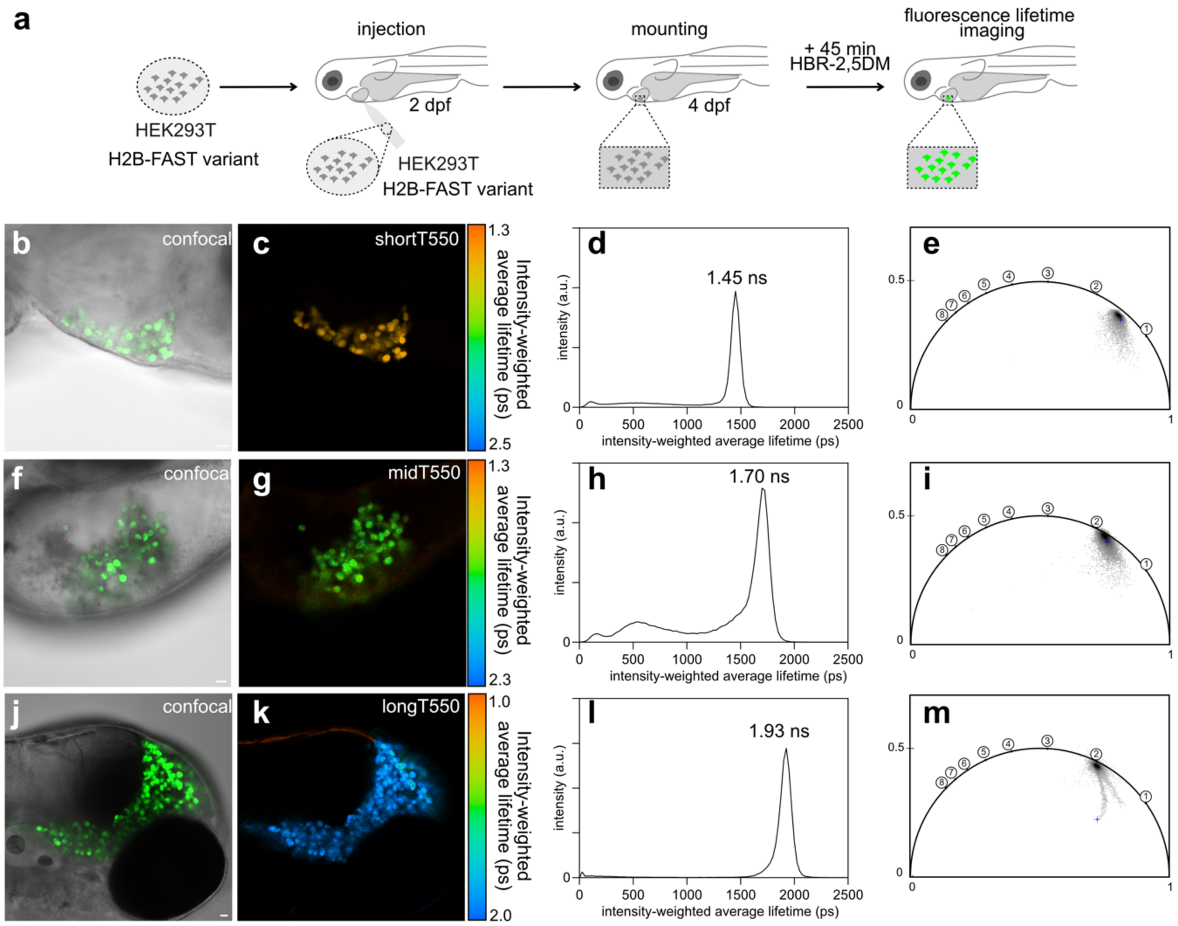

Figure S10. Fluorescence lifetime characterization of shortT550, midT550 and longT550 in zebrafish larvae. (a) Mammalian HEK293T were transfected with plasmid encoding either (**b-e**) H2B–shortT-FAST, (**f-i**) H2B–midT-FAST and (**j-m**) H2B–longT-FAST. After 24h, cell populations were injected either in the vitellus or in the developing brain of 2 dpf zebrafish larvae. Larvae were imaged at 4 dpf after 45 min incubation with 10 µM HBR-2,5DM. (**b,f,j**) Confocal micrographs. Scale bars, 20 µm. Excitation 488 nm / detection window 508-570 nm. (**c,g,k**) Intensity-weighted average lifetime color coded images. (**d,h,l**) Intensity-weighted lifetime histogram obtained by fitting the fluorescence decay data with a biexponential model. (**e,I,m**) Phasor plots of manually-drawn regions of interest around the injected cells (time in ns is shown on the universal circle). Phasor plot images were inverted using ezReverse (https://github.com/Morwey/ezreverse), an online app for inverting background.[9] Experiments were repeated three times with similar results.

Table S1. FAST variants properties with HBR-2,5DM

	K_D (μM)	λ_{abs} (nm)	ε ($mM^{-1}.cm^{-1}$)	λ_{em} (nm)	FQY (%)	T_i (ns)	k_r ($\times 10^8 s^{-1}$)	k_{nr} ($\times 10^8 s^{-1}$)
pFAST	0.01	498	52	549	0.30	1.79	1.68	3.91
greenFAST	0.12	494	54	551	0.25	1.34	1.87	5.60
oFAST	0.01	496	51	547	0.29	1.72	1.69	4.13
TsiA-FAST	0.01	498	52	549	0.34	1.91	1.78	3.46

Abbreviations are as follows: K_D thermodynamic dissociation constant, λ_{abs} wavelength of maximal absorption, ε molar absorptivity at λ_{abs} (standard error is typically 10%), λ_{em} wavelength of maximal emission, FQY fluorescence quantum yield. T_i intensity-weighted average fluorescence lifetime (in HEK293T cells), k_r radiative rate constant, k_{nr} non radiative rate constant. The constants k_r and k_{nr} were computed using the FQY and T_i values.

Table S2. FAST variants properties with HBR-3,5DM

	K_D (μM)	λ_{abs} (nm)	ε ($mM^{-1}.cm^{-1}$)	λ_{em} (nm)	FQY (%)	T_i (ns)	k_r ($\times 10^8 s^{-1}$)	k_{nr} ($\times 10^8 s^{-1}$)
pFAST	0.01	502	49	561	0.44	2.94	1.50	1.90
greenFAST	0.83	498	50	559	0.38	2.12	1.79	2.92
oFAST	0.014	502	48	561	0.43	2.98	1.44	1.91
TsiA-FAST	0.012	500	45	561	0.47	3.05	1.54	1.74

Abbreviations are as follows: K_D thermodynamic dissociation constant, λ_{abs} wavelength of maximal absorption, ε molar absorptivity at λ_{abs} (standard error is typically 10%), λ_{em} wavelength of maximal emission, FQY fluorescence quantum yield. T_i intensity-weighted average fluorescence lifetime (in HEK293T cells), k_r radiative rate constant, k_{nr} non radiative rate constant. The constants k_r and k_{nr} were computed using the FQY and T_i values.

Vector	ORF	ORF sequences
pAG109	H2B-FAST-cMyc	atgcccgaacctgcgaagtcagcgcccgctcccaaaaaaggctctaaaaaagctgtcgccaagacccagaagaaggggggataagaaaaggcgtaagaccaggaaagagagttacgccatttacgtgtacaaagtactaaaacaagtccacccggacactggcatctcctcaaaggcgatgggcattatgaactcatttgtaaacgacatcttcgagcgcatcgccggagaagcgtcgcgcctggcgcattacaacaagcgctccactatcacatcccgggagatccagacggccgtgcgcctgctcctgcccggagaactggccaaacacgctgtgtctgagggcacaaaggccgtgaccaagtacaccagctccaagggcggaggctccggaggcggatctgccaccatggagcatgttgcctttggcagtgaggacatcgagaacactctggccaaaatggacgacggacaactggatgggttggcctttggcgcaattcagctcgatggtgacgggaatatcctgcagtacaatgctgctgaaggagacatcacaggcagagatcccaaacaggtgattgggaagaacttcttcaaggatgttgcacctggaacggattctcccgagttttacggcaaattcaaggaaggcgtagcgtcagggaatctgaacaccatgttcgaatggatgataccgacaagcaggggaccaaccaaggtcaaggtgcacatgaagaaagccctttccggtgacagctattgggtctttgtgaaacgggtgggatccgaacaaaagcttatttctgaagaggacttg
pAG374	H2B-greenFAST-cMyc	atgcccgaacctgcgaagtcagcgcccgctcccaaaaaaggctctaaaaaagctgtcgccaagacccagaagaaggggggataagaaaaggcgtaagaccaggaaagagagttacgccatttacgtgtacaaagtactaaaacaagtccacccggacactggcatctcctcaaaggcgatgggcattatgaactcatttgtaaacgacatcttcgagcgcatcgccggagaagcgtcgcgcctggcgcattacaacaagcgctccactatcacatcccgggagatccagacggccgtgcgcctgctcctgcccggagaactggccaaacacgctgtgtctgagggcacaaaggccgtgaccaagtacaccagctccaagggcggaggctccggaggcggatctgccaccatggagcatgttgcctttggcagtgaggacatcgagaacactctggccaaaatggacgacgaacaactggatgggttggcctttggcgcaattcagctcgatggtgacgggaatatcctgcagtacaatgctgctgaaggagacatcacaggcagagatcccaaacaggtgattgggaagaacttcttcaaggatgttgcaactggaacggattctcccgagttttaccgcaaattcaaggaaggcgtagcgtcagggaatctgaacaccatgttcgaatggatgataccgacaagcaggggaccaaccaaggtcaaggtgcacatgaagaaagccctttccggtgacagctattgggtctttgtgaaacgggtgggatccgaacaaaagcttatttctgaagaggacttg
pAG472	H2B-iFAST-cMyc	atgcccgaacctgcgaagtcagcgcccgctcccaaaaaaggctctaaaaaagctgtcgccaagacccagaagaaggggggataagaaaaggcgtaagaccaggaaagagagttacgccatttacgtgtacaaagtactaaaacaagtccacccggacactggcatctcctcaaaggcgatgggcattatgaactcatttgtaaacgacatcttcgagcgcatcgccggagaagcgtcgcgcctggcgcattacaacaagcgctccactatcacatcccgggagatccagacggccgtgcgcctgctcctgcccggagaactggccaaacacgctgtgtctgagggcacaaaggccgtgaccaagtacaccagctccaagggcggaggctccggaggcggatctgccaccatggagcatgttgcctttggcagtgaggacatcgagaacactctggccaaaatggacgacggacaactggatgggttggcctttggcgcaattcagctcgatggtgacgggaatatcctgcagtacaatgctgctgaaggagacatcacaggcagagatcccaaacaggtgattgggaagaacttcttcaaggatgttgcacctggaacggattctcccgagttttacggcaaattcaaggaaggcgtagcgtcagggaatctgaacaccatgttcgaatggatgataccgacaagcaggggaccaaccaaggtcaagatacacatgaagaaagccctttccggtgacagctattgggtctttgtgaaacgggtgggatccgaacaaaagcttatttctgaagaggacttg

pAG657	H2B-pFAST-cMyc	atgcccgaacctgcgaagtcagcgcccgctcccaaaaaaggctctaaaaaagctgtc<br>gccaagacccagaagaaggggggataagaaaaggcgtaagaccaggaaagagagt<br>tacgccatttacgtgtacaaagtactaaaacaagtccacccggacactggcatctcctca<br>aaggcgatgggcattatgaactcatttgtaaacgacatcttcgagcgcatcgccggaga<br>agcgtcgcgcctggcgcattacaacaagcgctccactatcacatcccgggagatccag<br>acggccgtgcgcctgctcctgcccggagaactggccaaacacgctgtgtctgagggca<br>caaaggccgtgaccaagtacaccagctccaagggcggaggctccggaggcggatct<br>gccaccatggagcatgttgcctttggcagtgaggacatcgagaacactctggccaatat<br>ggacgacgaacaactggataggttggcctttggcgtaattcagctcgatggtgacggga<br>atatcctgctgtacaatgctgctgaaggggacatcactggcagagatcccaaacaggtg<br>attgggaagaacttcttcaaggatgttgcacctggaacggatactcccgagttttacggca<br>aattcaaggaaggcgcagcgtcagggaatctgaacaccatgttcgaatggacgatacc<br>gacaagcaggggaccaaccaaggtcaaggtgcacttgaagaaagccctttccggtga<br>cagatattgggtctttgtgaaacgggtgggatccgaacaaaagcttatttctgaagagga<br>cttg
pAG658	H2B-tFAST-cMyc	atgcccgaacctgcgaagtcagcgcccgctcccaaaaaaggctctaaaaaagctgtc<br>gccaagacccagaagaaggggggataagaaaaggcgtaagaccaggaaagagagt<br>tacgccatttacgtgtacaaagtactaaaacaagtccacccggacactggcatctcctca<br>aaggcgatgggcattatgaactcatttgtaaacgacatcttcgagcgcatcgccggaga<br>agcgtcgcgcctggcgcattacaacaagcgctccactatcacatcccgggagatccag<br>acggccgtgcgcctgctcctgcccggagaactggccaaacacgctgtgtctgagggca<br>caaaggccgtgaccaagtacaccagctccaagggcggaggctccggaggcggatct<br>gccaccatggagcatgttgcctttggcagtgaggacatcgagaacactctggccaaaat<br>ggacgacggacaactggataggttggcctttggcgcaattcagctcgatggtgacggg<br>aatatcctgaagtacaatgctgctgaaggagacatcacaggcagagatcccaaacag<br>gtgattgggaagaacttcttcaaggatgttgcacctggaacggatactcccgagttttacg<br>gcaaattcaaggaaggcgtatcgtcagggaatctgaacaccatgttcgaatgggcgat<br>accgacaagcaggggaccaaccaaggtcaaggtgcatttgaagaaagccctttccgg<br>tgacagatattgggtctttgtgaaacgggtgggatccgaacaaaagcttatttctgaagag<br>gacttg
pAG659	H2B-oFAST-cMyc	atgcccgaacctgcgaagtcagcgcccgctcccaaaaaaggctctaaaaaagctgtc<br>gccaagacccagaagaaggggggataagaaaaggcgtaagaccaggaaagagagt<br>tacgccatttacgtgtacaaagtactaaaacaagtccacccggacactggcatctcctca<br>aaggcgatgggcattatgaactcatttgtaaacgacatcttcgagcgcatcgccggaga<br>agcgtcgcgcctggcgcattacaacaagcgctccactatcacatcccgggagatccag<br>acggccgtgcgcctgctcctgcccggagaactggccaaacacgctgtgtctgagggca<br>caaaggccgtgaccaagtacaccagctccaagggcggaggctccggaggcggatct<br>gccaccatggagcatgttgcctttggcagtgaggacatcgagaacactctggccaaaat<br>ggacgacggacaactggatgggttggcctttggcgcaattcagctcgatggtgacggg<br>aatatcctgctgtacaatgctgctgaaggagacatcacaggcagagatcccaaacagg<br>tgattgggaagaacttcttcaaggatgttgcacctggaacgaattctcccgagttttacggc<br>aaattcaaggaaggcatagcgtcagggaatctgaacaccatgttcgaatggatgatac<br>cgacaagcaggggaccaaccaaggtcaaggtgcacttgaagaaagccctttccggtg<br>acagatattgggtctttgtgaaacgggtgggatccgaacaaaagcttatttctgaagagg<br>acttg
pAG1448	H2B-TsiA-FAST-cMyc	atgcccgaacctgcgaagtcagcgcccgctcccaaaaaaggctctaaaaaagctgtc<br>gccaagacccagaagaaggggggataagaaaaggcgtaagaccaggaaagagagt<br>tacgccatttacgtgtacaaagtactaaaacaagtccacccggacactggcatctcctca<br>aaggcgatgggcattatgaactcatttgtaaacgacatcttcgagcgcatcgccggaga<br>agcgtcgcgcctggcgcattacaacaagcgctccactatcacatcccgggagatccag<br>acggccgtgcgcctgctcctgcccggagaactggccaaacacgctgtgtctgagggca<br>caaaggccgtgaccaagtacaccagctccaagggcggaggctccggaggcggatct<br>gccaccatggagctgctgagcttcggcgccgacaacatcgagaacagcctggccaag<br>atgagcaagggcgacctgaacaagctggccttcggcgccatccagctgaacgcccag<br>ggcaagatcctgcagtacaacgccgccgagggcgacatcaccggcagaaagccca

		ccgaggtgatcggcaagaacttcttcctggaggtggcccccggcaccaacagaaccg<br>agttcaagggcagattcgaccagggcatcaagagcggcaacctgaacaccatgttcg<br>agtggatgatccccaccagcagaggccccaccaaggtgaaggtgcacatgaagaag<br>gccctggtggacgacacctactgggtgttcgtgaagagagtgggatccgaacaaaagc<br>ttatttctgaagaggacttg
pAG372	Mito-greenFAST-cMyc	atgtccgtcctgacgccgctgctgctgcggggcttgacaggctcggcccggcggctccc<br>agtgccgcgcgccaagatccattcgttgagatctgccaccatggagcatgttgcctttggc<br>agtgaggacatcgagaacactctggccaaaatggacgacgaacaactggatgggttg<br>gcctttggcgcaattcagctcgatggtgacgggaatatcctgcagtacaatgctgctgaa<br>ggagacatcacaggcagagatcccaaacaggtgattgggaagaacttcttcaaggat<br>gttgcaactggaacggattctcccgagttttaccgcaaattcaaggaaggcgtagcgtca<br>gggaatctgaacaccatgttcgaatggatgataccgacaagcaggggaccaaccaag<br>gtcaaggtgcacatgaagaaagccctttccggtgacagctattgggtctttgtgaaacgg<br>gtgggatccgaacaaaagcttatttctgaagaggacttg
pAG673	Mito-oFAST-cMyc	atgtccgtcctgacgccgctgctgctgcggggcttgacaggctcggcccggcggctccc<br>agtgccgcgcgccaagatccattcgttgagatctgccaccatggagcatgttgcctttggc<br>agtgaggacatcgagaacactctggccaaaatggacgacggacaactggatgggttg<br>gcctttggcgcaattcagctcgatggtgacgggaatatcctgctgtacaatgctgctgaag<br>gagacatcacaggcagagatcccaaacaggtgattgggaagaacttcttcaaggatgt<br>tgcacctggaacgaattctcccgagttttacggcaaattcaaggaaggcatagcgtcag<br>ggaatctgaacaccatgttcgaatggatgataccgacaagcaggggaccaaccaagg<br>tcaaggtgcacttgaagaaagccctttccggtgacagatattgggtctttgtgaaacgggt<br>gggatccgaacaaaagcttatttctgaagaggacttg
pAG667	MAP4-oFAST-cMyc	atggtgtcccggcaagaagaagcaaaggctgctgtaggtgtgactggaaatgacatca<br>ctaccccgccaaacaaggagccaccaccaagcccagaaaagaaagcaaagccttt<br>ggccaccactcaacctgcaaagacttcaacatcgaaagccaaaacacagcccacttc<br>tctccctaagcaaccagctcccaccacctctggtgggttgaataaaaaacccatgagcc<br>tcgcctcaggctcagtgccagctgccccacacaaacgccctgctgctgccactgctact<br>gccaggccttccaccctacctgccagagacgtgaagccaaagccaattacagaagct<br>aaggttgccgaaaagcggacctctccatccaagccttcatctgccccagccctcaaacc<br>tggacctaaaccaccccaaccgtttcaaaagccacatctccctcaactcttgtttccact<br>ggaccaagtagtagaagtccagctacaactctgcctaagaggccaaccagcatcaag<br>actgaggggaaacctgctgatgtcaaaaggatgactgctaagtctgcctcagctgacttg<br>agtcgctcaaagaccacctctgccagttctgtgaagagaaacaccactcccactgggg<br>cagcaccccccagcagggatgacttccactcgagtcaagcccatgtctgcacctagccg<br>ctcttctggggctctttctgtggacaagaagcccacttccactaagcctagctcctctgctcc<br>cagggtgagccgcctggccacaactgtttctgcccctgacctgaagagtgttcgctccaa<br>ggtcggctctacagaaaacatcaaacaccagcctggaggaggccgggccaaggtag<br>agaaaaaaacagaggcagctaccacagctgggaagcctgaacctaatgcagtcact<br>aaagcagccggctccattgcgagtgcacagaaaccgcctgctgggaaagtccagata<br>gtatccaaaaaagtgagctacagtcatattcaatccaagtgtgtttccaaggacaatatta<br>agcatgtccctggatgtggcaatgttcagattcagaacaagaaagtggacatatccaag<br>gtctcctccaagtgtgggtccaaagctaatatcaagcacaagcctggtggaggagatgt<br>caagattgaaagtcagaagttgaacttcaaggagaaggcccaagccaaagtgggag<br>gcggattcgcggatccaccggtcgccaccatgagtgtgattaaaccagacatggagca<br>tgttgcctttggcagtgaggacatcgagaacactctggccaaaatggacgacggacaa<br>ctggatgggttggcctttggcgcaattcagctcgatggtgacgggaatatcctgctgtaca<br>atgctgctgaaggagacatcacaggcagagatcccaaacaggtgattgggaagaact<br>tcttcaaggatgttgcacctggaacgaattctcccgagttttacggcaaattcaaggaagg<br>catagcgtcagggaatctgaacaccatgttcgaatggatgataccgacaagcagggg<br>accaaccaaggtcaaggtgcacttgaagaaagccctttccggtgacagatattgggtctt<br>tgtgaaacgggtgggatccgaacaaaagcttatttctgaagaggacttg
	HboL-FAST	atggagaccgtgagattcggcggcgacgacatcgagaacagcctggccaagatgga<br>cgacaagaagctggacgagctggccttcggcgccatccagctggacgccaacggca<br>agatcatccagtacaacgccgccgagggcggcatcaccggcagagaccccaagag

		cgtgatcggcaagaacttcttcaccgaggtggcccccggcacccagagcaaggagttc<br>cagggcagattcaaggagggcgtgagcagcggcgagctgaacaccatgttcgagtg<br>gatgatccccaccagcagaggccccaccaaggtgaaggtgcacatgaagaaggcc<br>atcagcggcgacacctactggatcttcgtgaagagactg
	HspG-FAST	atggagaccgtgagattcggcggcgacgacatcgagaacgccctggccaacatgga<br>cgacaagaagctggacaccctggccttcggcgccatccagctggacgccaacggca<br>agatcatccagtacaacgccgccgagggcggcatcaccggcagagaccccaagag<br>cgtgatcggcaagaacttcttcaccgacgtggcccccggcacccagagcaaggagttc<br>cagggcagattcaaggagggcgtgaagaacggcgacctgaacaccatgttcgagtg<br>gatgatccccaccagcagaggccccaccaaggtgaaggtgcacatgaagaaggccc<br>tgagcggcgacaccttctggatcttcgtgaagagactg
	RspA-FAST	atggagaccgtgagattcggcggcgacgacatcgagaacagcctggccaagatgga<br>cgacaaggccctggacaagctggccttcggcgccatccagctggacggcaacggca<br>agatcatccactacaacgccgccgagggcaccatcaccggcagagaccccaagacc<br>gtgatcggcaagaacttcttcaccgacgtggcccccggcacccagagcaaggagttcc<br>agggcagattcaaggagggcgtgcagaagggcgacctgaacaccatgttcgagtgg<br>atgatccccaccagcagaggccccaccaaggtgaaggtgcacatgaagaaggccat<br>gaccggcgacagcttctggatcttcgtgaagagactg
	Ilo-FAST	atggagatcgtgcagttcggcagcgacgacatcgagaacaccctgagcaagatgagc<br>gacgacaagctgaacgacatcgccttcggcgccatccagctggacgccagcggcaa<br>gatcatccagtacaacgccgccgagggcgacatcaccggcagagaccccggcgcc<br>gtggtgggcaagaacttcttcaacgaggtggcccccggcaccaacagccccgagttca<br>agggcagattcgacgagggcgtgaagaacggcaacctgaacaccatgttcgagtgg<br>atgatccccaccagcagaggccccaccaaggtgaaggtgcacatgaagaaggccct<br>gaccggcgacacctactgggtgttcgtgaagagactg
	Rsa-FAST	atggagatgatcaagttcggccaggacgacatcgagaacgccatggccgacatgggc<br>gacgcccagatcgacgacctggccttcggcgccatccagctggacgagaccggcacc<br>atcctggcctacaacgccgccgagggcgagctgaccggcagaagcccccaggacgt<br>gatcggcaagaacttcttcaaggacatcgcccccggcaccgacaccgaggagttcgg<br>cggcagattcagagagggcgtggccaacggcgacctgaacgccatgttcgagtggat<br>gatccccaccagcagaggccccaccaaggtgaaggtgcacatgaagagagccatca<br>ccggcgacagctactggatcttcgtgaagagagtg
pAG1367	H2B-emIRFP670-cMyc	atgcccgaacctgcgaagtcagcgcccgctcccaaaaaaggctctaaaaaagctgtc<br>gccaagacccagaagaagggggataagaaaaggcgtaagaccaggaaagagagt<br>tacgccatttacgtgtacaaagtactaaaacaagtccacccggacactggcatctcctca<br>aaggcgatgggcattatgaactcatttgtaaacgacatcttcgagcgcatcgccggaga<br>agcgtcgcgcctggcgcattacaacaagcgctccactatcacatcccgggagatccag<br>acggccgtgcgcctgctcctgcccggagaactggccaaacacgctgtgtctgagggca<br>caaaggccgtgaccaagtacaccagctccaagggcggaggctccggaggcggatct<br>gccaccatggcggaaggatccgtcgccaggcagcctgacctcttgacctgcgaacatg<br>aagagatccacctcgccggctcgatccagccgcatggcgcgcttctggtcgtcagcga<br>acatgatcatcgcgtcatccaggccagcgccaacgccgcggaatttctgaatctcggaa<br>gcgtactcggcgttccgctcgccgagatcgacggcgatctgttgatcaagatcctgccgc<br>atctcgatcccaccgccgaaggcatgccggtcgcggtgcgctgccggatcggcaatcc<br>ctctacggagtactgcggtctgatgcatcggcctccggaaggcgggctgatcatcgaac<br>tcgaacgtgccggcccgtcgatcgatctgtcaggcacgctggcgccggcgctggagcg<br>gatccgcacggcgggttcactgcgcgcgctgtgcgatgacaccgtgctgctgtttcagca<br>gtgcaccggctacgaccgggtgatggtgtatcgtttcgatgagcaaggccacggcctgg<br>tattctccgagtgccatgtgcctgggctcgaatcctatttcggcaaccgctatccgtcgtcg<br>actgtcccgcagatggcgcggcagctgtacgtgcggcagcgcgtccgcgtgctggtcg<br>acgtcacctatcagccggtgccgctggagccgcggctgtcgccgctgaccggcgcg<br>atctcgacatgtcgggctgcttcctgcgctcgatgtcgccgtgccatctgcagttcctgaag<br>gacatgggcgtgcgcgccaccctggcggtgtcgctggtggtcggcggcaagctgtggg<br>gcctggttgtctgtcaccattatctgccgcgcttcatccgtttcgagctgcgggcgatctgca

		aacggctcgccgaaaggatcgcgacgcggatcaccgcgcttgagagcggatccgaa caaaagcttatttctgaagaggacttg

EXPERIMENTAL SECTION

General Commercially available reagents were used as obtained. The synthesis of HBR-2,5DM,[2] HBR-3,5DM[2] and HMBR[1] was previously reported. These fluorogens are commercially available from the Twinkle Factory under the name $match_{550}$, ^{TF}Amber and ^{TF}Lime.

Biology. The presented research complies with all relevant ethical regulations.

General Synthetic oligonucleotides used for cloning were purchased from Integrated DNA Technology. PCR reactions were performed with Q5 polymerase (New England Biolabs) in the buffer provided. PCR products were purified using QIAquick PCR purification kit (QIAGEN). DNase I, T4 ligase, fusion polymerase, Taq ligase and Taq exonuclease were purchased from New England Biolabs and used with accompanying buffers and according to the manufacturer’s protocols. Isothermal assemblies (Gibson Assembly) were performed using a homemade mix prepared according to previously described protocols.[10] Small-scale isolation of plasmid DNA was conducted using a QIAprep miniprep kit (QIAGEN) from 2 mL overnight bacterial culture supplemented with appropriate antibiotics. Large-scale isolation of plasmid DNA was conducted using the QIAprep maxiprep kit (QIAGEN) from 150 mL overnight bacterial culture supplemented with appropriate antibiotics. All plasmid sequences were confirmed by Sanger sequencing with appropriate sequencing primers (GATC Biotech).

Cloning The plasmids used in this study have been generated using isothermal Gibson Assembly or restriction enzymes cloning.

The plasmids pAG641, pAG261, pAG382 and pAG645 enabling the bacterial expression of 6 ×His–TEVcs–pFAST,[7] 6 ×His–TEVcs–greenFAST,[6] 6 ×His–TEVcs–TsiA-FAST,[8] and 6 ×His–TEVcs–oFAST[7] were previously described.

The construction of plasmids pAG109 pAG374, pAG472, pAG657, pAG658 and pAG659 allowing the mammalian expression of H2B-FAST-cMyc,[1] H2B-greenFAST-cMyc,[6] H2B-iFAST-cMyc,[5] H2B-pFAST-cMyc,[7] H2B-tFAST-cMyc[7] and H2B-oFAST-cMyc[7] was previously described. The construction of plasmids pAG372 pAG673 and pAG665 allowing the expression of mito-greenFAST-cMyc,[6] mito-oFAST-cMyc[7] and MAP4-oFAST-cMyc[7] was

previously described. The plasmids pAG765, pAG766, pAG767, pAG768, pAG769 and pAG770 allowing the expression of cMyc-HboL-FAST-IRES-mTurquoise2, cMyc-HspG-FAST-IRES-mTurquoise2, cMyc-RspA-FAST-IRES-mTurquoise2, cMyc-Ilo-FAST-IRES-mTurquoise2, cMyc-TsiA-FAST-IRES-mTurquoise2, cMyc-Rsa-FAST-IRES-mTurquoise2 were previously described[8]. The plasmids pAG1367 and pAG1448 allowing the mammalian expression of H2B-emiRFP670-cMyc and H2B-TsiA-FAST-cMyc respectively were constructed by replacing the sequence of pFAST by the sequence of emiRFP670 (Addgene 136556) and TsiA-FAST in the plasmid pAG657 allowing the expression of H2B-pFAST-cMyc. The plasmids pAG324 and pAG1247 allowing the expression of H2B-mCherry[11] and H2B-ECFP-pFAST$_{1\text{-}114}$[12] were previously described. The plasmid pAG1052 allowing the expression of FRB-EGFP was obtained by replacing the sequence of frFAST$_{1\text{-}114}$ by the sequence of EGFP in the plasmid pAG499 allowing the expression of CMV-cMyc-FRB-N-frFAST.[11]

Cell culture HeLa cells (ATCC CRM-CCL2) were cultured in minimal essential medium supplemented with phenol red, Glutamax I, 1 mM of sodium pyruvate, 1% (vol/vol) of non-essential amino acids, 10% (vol/vol) fetal calf serum (FCS) and 1% (vol/vol) penicillin–streptomycin at 37 °C in a 5% CO_2 atmosphere. HEK293T (ATCC CRL-3216) cells were cultured in Dulbecco's modified Eagle medium (DMEM) supplemented with phenol red, 10% (vol/vol) FCS and 1% (vol/vol) penicillin–streptomycin at 37 °C in a 5% CO_2 atmosphere. U2OS cells (ATCC HTB-96) were cultured in McCoy's medium supplemented with phenol red and 10% (vol/vol) FCS and 1% (vol/vol) penicillin–streptomycin at 37 °C in a 5% CO_2 atmosphere. For imaging, cells were seeded in µDish IBIDI (Biovalley) coated with poly-L-lysine. Cells were transiently transfected using Genejuice (Merck) according to the manufacturer's protocols for 24-48 h before imaging. Cells were washed with Dulbecco's PBS (DPBS) and treated with DMEM (without serum and phenol red) supplemented with the compounds at the indicated concentration.

Protein expression in bacteria and purification Plasmids were transformed in BL21(DE3) competent *Escherichia coli* (New England Biolabs) or Rosetta(DE3)pLysS *E. coli* (Merck). Cells were grown at 37 °C in lysogeny broth medium supplemented with 50 µg.mL^{-1} kanamycin (and 34 µg.mL^{-1} of chloramphenicol for Rosetta) to OD_{600nm} 0.6. Expression was induced overnight at 16 °C by adding isopropyl β-D-1-thiogalactopyranoside (IPTG) to a final concentration of 1 mM. Cells were collected by centrifugation (4,300 × g for 20 min at 4 °C) and frozen. For purification, the cell pellet was resuspended in lysis buffer (PBS supplemented with 2.5 mM $MgCl_2$, 1 mM of protease inhibitor phenylmethanesulfonyl fluoride and 0.025 mg.mL^{-1} DNase, pH 7.4) and sonicated (5 min, 20% of amplitude) on ice. The lysate was incubated for 2 h on ice to allow DNA digestion by DNase. Cellular fragments were removed by centrifugation

(9,000 × g for 1 h at 4 °C). The supernatant was incubated overnight at 4 °C by gentle agitation with pre-washed Ni-NTA agarose beads in PBS buffer complemented with 20 mM of imidazole. Beads were washed with ten volumes of PBS complemented with 20 mM of imidazole and with five volumes of PBS complemented with 40 mM of imidazole. His-tagged proteins were eluted with five volumes of PBS complemented with 0.5 M of imidazole. The buffer was exchanged to PBS (0.05 M phosphate buffer and 0.150 M NaCl) using PD-10 desalting columns or Midi-Trap G-25 (GE Healthcare). The purity of the proteins was evaluated using SDS–PAGE electrophoresis stained with Coomassie blue.

Physicochemical measurements Steady-state UV-Vis and fluorescence spectra were recorded at 25 °C on a Spark 10 M (Tecan). Data were processed using GraphPad Prism v.10.0.3. Fluorescence quantum yield measurements were determined in 96-well plates using either FAST:HMBR or pFAST:HBR-3,5DM as a reference. Solutions of 40 µM of proteins were used, in which the fluorogen was diluted to the right concentration, usually from 6 µM to 0.375 µM, allowing > 99% of complex formation. Absorption coefficients were determined directly by the previous experiments after determination of the optical path length. Thermodynamic dissociation constants were determined by titration experiments in which we measured the fluorescence of the fluorescent assembly at various fluorogen concentrations using a Spark 10M plate reader (Tecan) and fitting data in Prism 9 to a one-site specific binding model.

Fluorescence microscopy The confocal micrographs were acquired on a Zeiss LSM 980 Laser Scanning Microscope equipped with a plan apochromat 20 × dry (NA 0.8) objective and with a plan apochromat 63× /1.4 NA oil immersion objective ZEN software were used to collect the confocal data. Fiji was used to analyze the confocal data.

Fluorescence lifetime imaging

General FLIM images were acquired on a Zeiss LSM 980 confocal system coupled with Becker and Hickl FLIM module and equipped with a 20 × dry (NA 0.8) objective or a 63 × oil immersion (NA 1.4) objective. A 488 nm pulsed diode laser (50 MHz) for the excitation, a green large band bloc filter (DBS 450 + 538; DBP 467/24 + 598/110) and an HPM-100-40-ZEISS Hybrid GaAsP Photodetector were used. Acquisitions were performed at 37°C for mammalian cells and 30°C for zebrafish. All FLIM analysis was performed on the software SPCImage of Becker and Hickl.[13] A binning of n = 1 was set for data analysis.

Theoretical background A detailed description of the theoretical background used for data analysis can be found in reference [13]. Hereafter is a short description of the essential. In time-correlated single photon counting FLIM (TCSPC-FLIM), a short pulse is used for excitation of the fluorescent molecules using a high frequency pulsed laser. The sample is

scanned, and each pixel of the image corresponds to the fluorescence decay curve I(t) obtained from the temporal photon distribution.

Fitting approaches

The fluorescence decay of an emissive species can be modelled by a monoexponential distribution:

$$I(t) = \alpha \exp(-\frac{t}{T})$$

where I(t) is the measured signal as a function of time t, α is the amplitude, and T is the fluorescence lifetime.

In some cases, the fluorescence decay is better fitted with a multicomponent exponential function:

$$I(t)=\sum_{k=1}^{n} \alpha_k \exp\left(-\frac{t}{T_k}\right)$$

where n indicates the total number of components, and α_k and T_k the amplitude and lifetime of component k, respectively. In the case of a multi-exponential decay, we define the intensity-weighted average fluorescence lifetime T_i as:

$$T_i = \frac{\int_0^\infty t\, I(t)dt}{\int_0^\infty I(t)dt} = \frac{\int_0^\infty t \sum_{k=1}^{n} \alpha_k \exp(-\frac{t}{T_k})dt}{\int_0^\infty \sum_{k=1}^{n} \alpha_k \exp(-\frac{t}{T_k})dt} = \frac{\sum_{k=1}^{n} \alpha_k {T_k}^2}{\sum_{k=1}^{n} \alpha_k T_k}$$

As the T_i corresponds mathematically to the intensity-weighted arithmetic average of the different lifetimes, it can be used as a parameter to compare different fluorescent systems, no matter if their fluorescence decays are best fitted using single, bi or triexponential models.

Associated with the decays are the residuals as well as reduced χ^2 parameter. These two parameters allow the evaluation of whether a model is well suited for a set of decay data. The residuals measure the differences between the measured decay values and the corresponding fit function. The reduced χ^2 is an indicator of the goodness of the fit. The closer this value gets to 1, the better the fit. We used the maximum-likelihood estimation (MLE) as a fit algorithm.

Non-fitting phasor plot approach

The non-fitting phasor plot approach allows a visual representation in polar coordinates of the different fluorescent decays of the species present in a sample: pixels with similar fluorescence decays will result in a cluster on the phasor plot, and it is possible to reciprocally identify regions

of the image based on the positions of the pixels on the phasor plot. Decay from a pixel with (x,y) positions in the image, noted $I_{x,y}(t)$ is converted using a Fourier transform into phasor coordinates $g_{x,y}(\omega)$ and $s_{x,y}(\omega)$ defined as :

$$g_{x,y}(\omega)=\frac{\int_0^\infty I_{x,y}(t)\cos(\omega t)dt}{\int_0^\infty I_{x,y}(t)dt}$$

and:

$$s_{x,y}(\omega)=\frac{\int_0^\infty I_{x,y}(t)\sin(\omega t)dt}{\int_0^\infty I_{x,y}(t)dt}$$

The phasor plot does not require any assumptions on the number of components required for fitting a decay. However, species showing single exponential decays result in a cluster situated on the universal circle. Species giving cluster inside the universal circle are considered having multi-component exponential decays.

Single variants characterization For each variant, at least five to six fields of view using a 20 × objective from two biological replicates were acquired, first through confocal imaging then through lifetime imaging. For accurate lifetime determination, a number of photons per pixel between 100 and 1000 (with an average of about 300 photons per pixel) was collected. Regions of interest were drawn around the nuclei, and the corresponding fluorescence decays were fitted with monoexponential or biexponential models (using maximum likelihood estimation (MLE) for better accuracy). For each fitting model, the fluorescence lifetime components were reported, and the intensity-weighted average lifetime was computed. For each FAST:fluorogen assembly, > 70 cells from two biological replicates were analyzed this way. The mean of intensity-weighted average fluorescence lifetime was used as a parameter to characterize the fluorescent assemblies.

Fluorescence lifetime multiplexing For each pair or triplet, confocal micrographs using 63 × objective were acquired prior to lifetime imaging to ensure optimal focus and thus better data quality. After lifetime acquisition, separation of the two or three variants was performed systematically through:

(i) Calculation of the first moment: this gives a distribution of average photon arrival time. Variants that can be separated based on their lifetimes appear in different colors.

(ii) Fitting of the fluorescence decays with biexponential model and plotting of the corresponding intensity-weighted lifetime distribution through the whole image. Variants that can be separated will result in two distinct peaks in the resulting

histogram and selection of lifetime windows around the peaks enables separation of the two variants.

(iii) After drawing a region of interest around the cell to analyze, the corresponding phasor plot can be displayed, and pixels with similar lifetimes form a cluster. Two distinct clusters are visible when the variants can be separated. Pixels forming each cluster can be identified in the lifetime image and thus separated.

As above, for accurate lifetime determination, a number of photons per pixel between 100 and 1000 (with an average of about 300 photons per pixel) were collected. Note that for the experiments involving co-expression of several proteins, expression levels were adjusted to obtain similar brightness in cells to facilitate photon counting and fluorescence lifetime determination. This rule is not fully mandatory if photon collection is sufficient for extracting reliably a fluorescence lifetime but does not saturate detectors. We report in Annex the quantification of the intracellular brightness for the different intensity images we show in this paper. We estimate from our experiments that as long as the cellular brightness of two species are not more than fivefold different, it is possible to reliably quantify their fluorescence lifetimes.

Zebrafish experiments

Adult zebrafish (*Danio rerio*) were kept at around 27-29°C on a 14 hr-light: 10 hr-dark cycle and fed twice daily. Natural crosses obtained fertilized eggs which were raised at 28°C in Volvic water. Experiments were performed using the standard nacre strain. Developmental stages were determined and indicated as days post fertilization (dpf). The animal facility obtained permission from the French Ministry of Agriculture (agreement No. D-75-05-32.) and all animal procedures were performed in accordance with French animal welfare guidelines. HEK293T were seeded at 500,000 cells/mL concentration in 25 cm^2 flasks and transfected after 24h using GeneJuice (Merck) according to manufacturer's guidelines. After 24h, cells were harvested at 10,000,000 cells/mL concentration in serum free DMEM. Cell suspension was loaded into a borosilicate glass needle pulled by a Flaming/Brown micropipette puller (Narishige, Japan, PN-30). 5~10 nanoliters suspension were implanted into anesthetized (0.02% MS-222 tricaine (Sigma)) 2 dpf zebrafish larvae within the developing brain by using an electronically regulated air-pressure microinjector (FemtoJet, Eppendorf). After injection, zebrafish larvae were placed in Volvic water and examined under a stereoscopic microscope for the presence of fluorescent cells and then raised for two more days at 28°C before imaging. For imaging, living zebrafish larvae were anesthetized in MS-222 tricaine solution and embedded in a lateral orientation in low-melting agarose (0.8%). Larval zebrafish were studied before the onset of sexual differentiation and their sex can therefore not be determined.

ANNEX

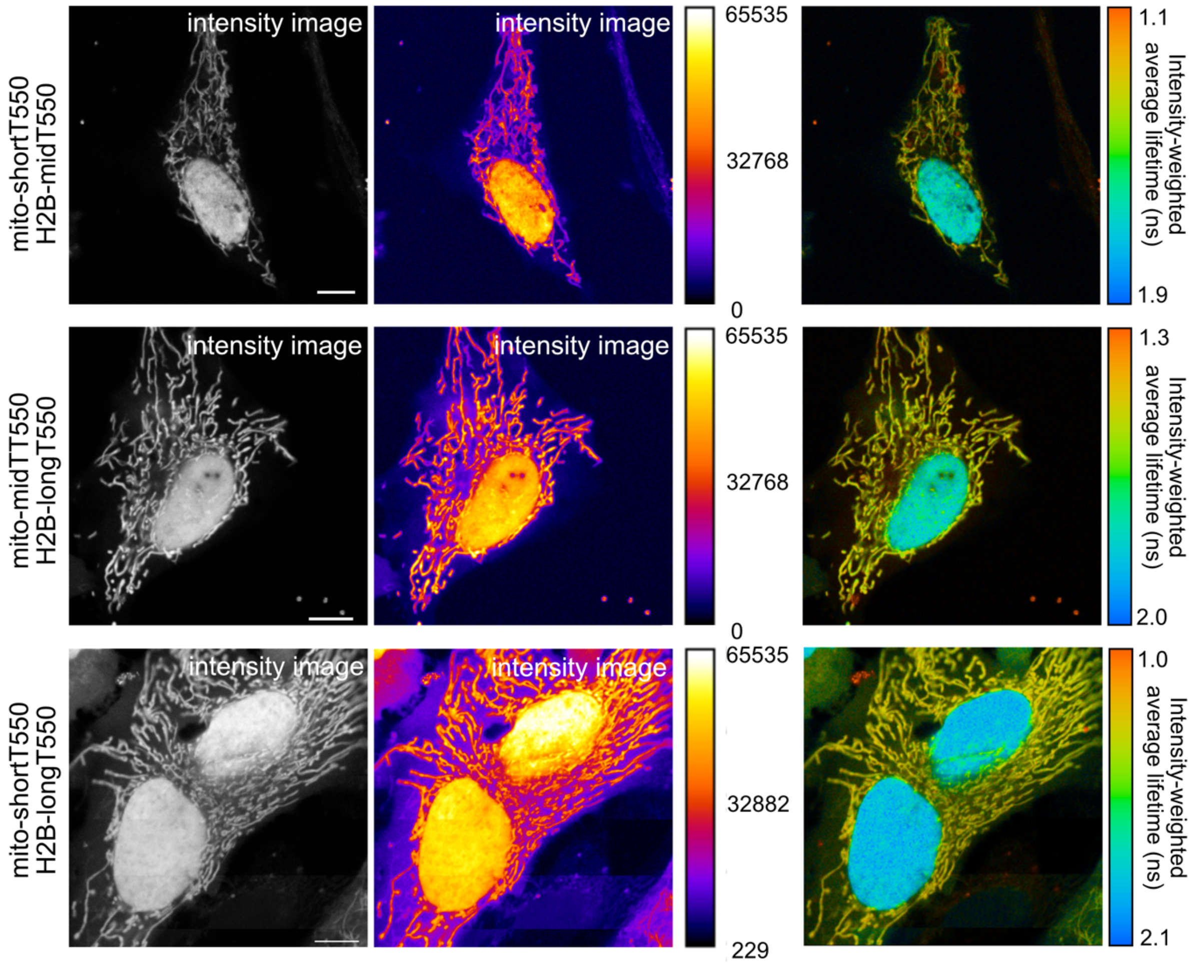

Annex Figure 1. Addition to Figure 2. This figure shows the intensity images shown in Figure 2 in color-coded mode (images in the middle) in order to better appreciate the relative brightness of the fluorescent species present.

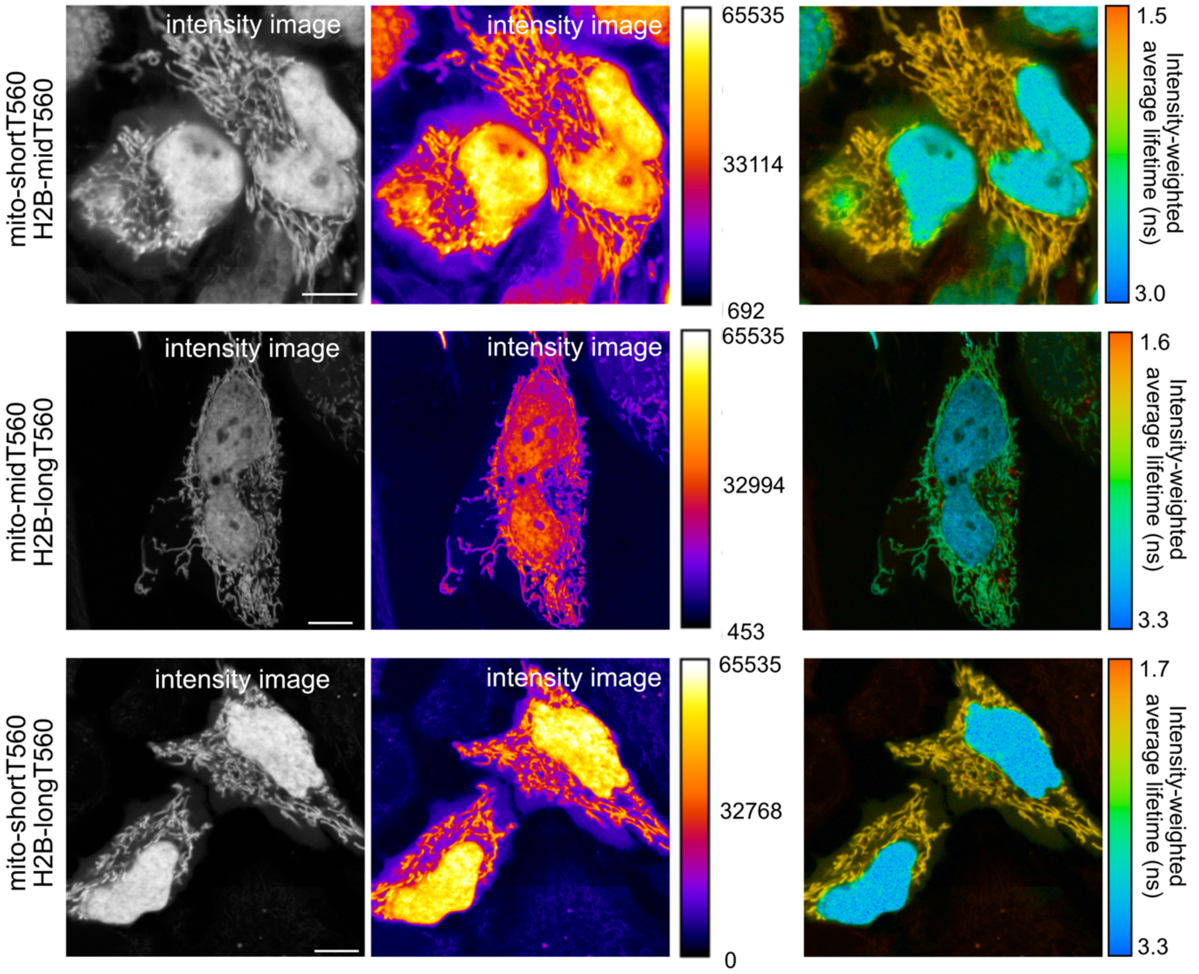

Annex Figure 2. Addition to Figure 3. This figure shows the intensity images shown in Figure 3 in color-coded mode (images in the middle) in order to better appreciate the relative brightness of the fluorescent species present.

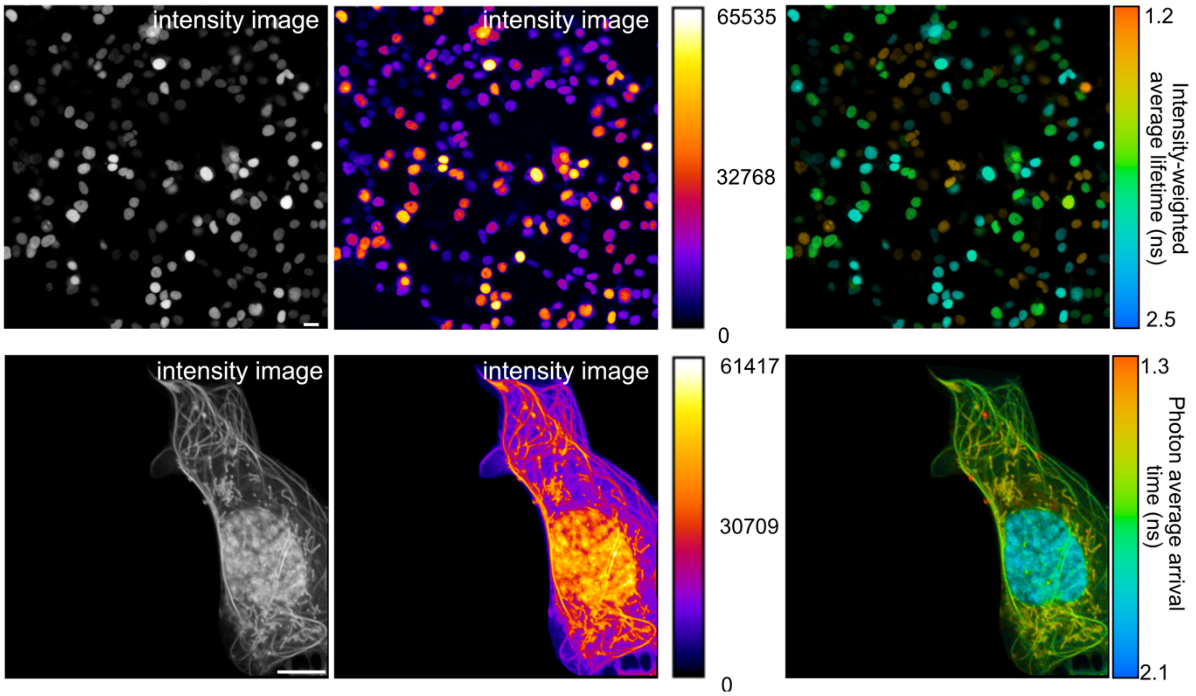

Annex Figure 3. Addition to Figure 4. This figure shows the intensity images shown in Figure 4 in color-coded mode (images in the middle) in order to better appreciate the relative brightness of the fluorescent species present.

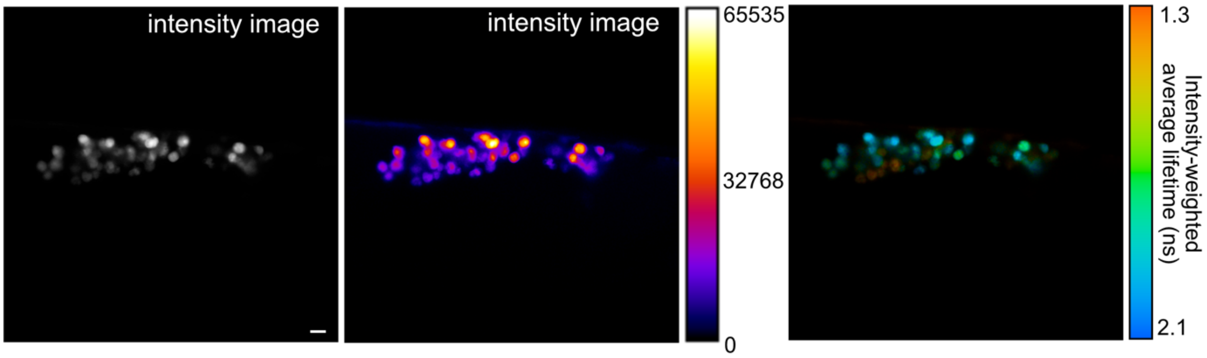

Annex Figure 4. Addition to Figure 5. This figure shows the intensity image shown in Figure 5 in color-coded mode (image in the middle) in order to better appreciate the relative brightness of the fluorescent species present.

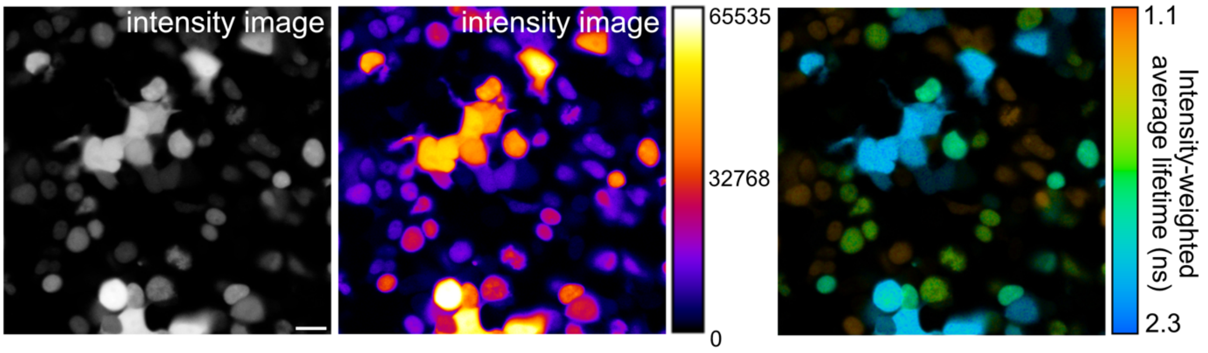

Annex Figure 5. Addition to Figure S9. This figure shows the intensity image shown in Figure S9 in color-coded mode (image in the middle) in order to better appreciate the relative brightness of the fluorescent species present.

REFERENCES

[1] M. A. Plamont, E. Billon-Denis, S. Maurin, C. Gauron, F. M. Pimenta, C. G. Specht, J. Shi, J. Quérard, B. Pan, J. Rossignol, N. Morellet, M. Volovitch, E. Lescop, Y. Chen, A. Triller, S. Vriz, T. Le Saux, L. Jullien, A. Gautier, *Proc Natl Acad Sci U S A* **2016**, *113*, 497–502.

[2] C. Li, M. A. Plamont, H. L. Sladitschek, V. Rodrigues, I. Aujard, P. Neveu, T. Le Saux, L. Jullien, A. Gautier, *Chem Sci* **2017**, *8*, 5598–5605.

[3] A. F. Philip, K. T. Eisenman, G. A. Papadantonakis, W. D. Hoff, *Biochemistry* **2008**, *47*, 13800–13810.

[4] A. F. Philip, R. A. Nome, G. A. Papadantonakis, N. F. Scherer, W. D. Hoff, *Proc Natl Acad Sci U S A* **2010**, *107*, 5821–5826.

[5] A. G. Tebo, F. M. Pimenta, Y. Zhang, A. Gautier, *Biochemistry* **2018**, *57*, 5648–5653.

[6] A. G. Tebo, B. Moeyaert, M. Thauvin, I. Carlon-Andres, D. Böken, M. Volovitch, S. Padilla-Parra, P. Dedecker, S. Vriz, A. Gautier, *Nat Chem Biol* **2021**, *17*, 30–38.

[7] H. Benaissa, K. Ounoughi, I. Aujard, E. Fischer, R. Goïame, J. Nguyen, A. G. Tebo, C. Li, T. Le Saux, G. Bertolin, M. Tramier, L. Danglot, N. Pietrancosta, X. Morin, L. Jullien, A. Gautier, *Nat Commun* **2021**, *12*, 6989.

[8] L. M. Rakotoarison, A. G. Tebo, D. Böken, S. Board, L. El Hajji, A. Gautier, *ACS Chem Biol* **2024**, *19*, 428–441.

[9] X. Song, J. Goedhart, *bioRxiv* **2024**, 2024.05.27.594095.

[10] D. G. Gibson, L. Young, R. Y. Chuang, J. C. Venter, C. A. Hutchison, H. O. Smith, *Nat Methods* **2009**, *6*, 343–345.

[11] C. Li, A. G. Tebo, M. Thauvin, M. Plamont, M. Volovitch, X. Morin, S. Vriz, A. Gautier, *Angewandte Chemie* **2020**, *132*, 18073–18079.

[12] S. Bottone, O. Joliot, Z. V. Cakil, L. El Hajji, L. M. Rakotoarison, G. Boncompain, F. Perez, A. Gautier, *Nat Methods* **2023**, *20*, 1553–1562.

[13] W. Becker, The bh TCSPC handbook. 10th edition **2023**, available on www.becker-hickl.com
